# Nano-MIL-88A(Fe) Enabled Clear Cellulose Films with Excellent UV-Shielding Performance and Robust Environment Resistance

**DOI:** 10.3390/nano12111891

**Published:** 2022-05-31

**Authors:** Lijian Sun, Xianhui An, Xueren Qian

**Affiliations:** Key Laboratory of Bio-Based Material Science & Technology, Northeast Forestry University, Ministry of Education, Harbin 150040, China; lantian0308@nefu.edu.cn (L.S.); anxianh509@163.com (X.A.)

**Keywords:** cellulose film, nano-MIL-88A(Fe), high transmittance, low haze, UV-blocking, environment resistance

## Abstract

While tremendous efforts have been dedicated to developing cellulose-based ultraviolet (UV)-blocking films, challenges still remain in simultaneously achieving high transparency, low haze and excellent UV shielding properties via simple and green strategy. Here, we present a facile and eco-friendly route to fabricate flexible, biodegradable and clear UV-shielding nano-MIL-88A(Fe)@carboxymethylated cellulose films (M(Fe)CCFs) via in situ synthesis of nano-MIL-88A(Fe) in carboxymethylated cellulose hydrogel followed by natural drying. The carboxymethylated cellulose film has high transmittance (93.2%) and low haze (1.8%). The introduction of nano-MIL-88A(Fe) endowed M(Fe)CCFs superior UV-shielding ability, while retaining high transmittance (81.5–85.3%) and low haze (2.5–4.9%). Moreover, M(Fe)CCFs showed stable UV blocking performance under UV irradiation, high temperature, acidic or alkaline conditions. Quite encouragingly, the UV-shielding ability of M(Fe)CCFs did not deteriorate, even after 30 days of immersion in aqueous solution, providing films with a long-term use capacity. Thus, M(Fe)CCFs show high potential in the UV protection field. Overall, these UV-blocking films with outstanding performances are a promising candidate to replace conventional film materials made from synthetic polymers in fields such as packaging and flexible electronics.

## 1. Introduction

In recent decades, climate change and environmental pollution caused by human activities have resulted in serious damage to the earth’s ozone layer, and the amount of ultraviolet (UV) light reaching the Earth’s surface has increased significantly, causing a series of issues, i.e., sunburn, skin cancer, yellowing of paper and discoloration of dyes, and other problems associated with UV light [1,2,3]. For these reasons, emphasis has been given toward the development of UV blocking products, such as visually transparent coating for UV-sensitive materials or UV protective films. The synthetic polymer matrices derived from petrochemicals have been widely used in UV blocking materials. However, such materials used in UV shielding products for specific applications contain non-biodegradable substances [4,5]. The disposal of synthetic polymer waste generates a great number of hazardous and toxic substances, which can seriously damage the environment. As a result, with growing concern over environmental problems and the overexploitation of fossil resources, cellulose as a kind of renewable and biodegradable natural polymer in nature has been developed to replace traditional synthetic polymer matrices [6,7,8]. In particular, cellulose-based film materials show high transparency, thermal stability and excellent mechanical performance [9]. Compared to plastic substrates, cellulose film materials have a much lower coefficient of thermal expansion and can also withstand a much higher processing temperature [10]. Therefore, from an environmental perspective, cellulose films are expected to have great potential to replace traditional synthetic polymer matrices in UV blocking applications. The general approach to obtain high UV shielding capability relies on the incorporation of UV absorbers into the cellulose matrix. However, the uniform distribution of UV absorbers in the cellulose matrix remains a major problem in the fabrication of UV-blocking films, mainly because the uneven distribution of the UV absorber leads to a decrease in visible light transmittance, in addition to an increase in haze, which affects the practical application of UV-blocking materials.

The modification of cellulosic materials using organic derivatives and inorganic nano/micron oxides (e.g., TiO_2_, ZnO, Al_2_O_3_ and CeO_2_) has become a very important approach to realize UV shielding functions [11,12,13,14]. However, the use of these UV-absorbing materials is also accompanied by some serious issues, manifested in difficulties of the preparation process, which includes many steps, high chemical consumption, and the use of hazardous solvents and toxic chemicals [15]. Furthermore, UV absorption of inorganic nano/micron oxides between 200–400 nm is incomplete due to their inherent wide band gap. Meanwhile, the mismatch between the refractive indices and the incompatibility between these inorganic absorbers and the polymer matrix also cause lower transparency of the polymer matrix. Recently, organic-inorganic hybrid material, namely metal-organic frameworks (MOFs), has also been used as a UV absorber to prepare UV shielding materials [15,16,17,18,19,20]. In contrast to semiconductor nanoparticles such as ZnO and TiO_2_, the tunability of the structure allows the design of UV shielding functions to be conferred on the assembled MOFs materials. MOF materials inevitably also have some drawbacks, such as the need for high temperature and pressure synthesis and the use of toxic solvents. In view of these problems, there is an urgent need to find MOF materials with efficient UV shielding capacity that can be synthesized by a simple, room temperature, green method and then used to prepare cellulose-based UV protective films.

Iron is a transition metal with UV photoprotective properties. Researchers have verified the protective effect of iron against UV radiation [21]. Considering that the absorption of Fe^3+^ occurs in the near-UV range of the electromagnetic spectrum, Fe^3+^ is used as a UV absorber and can be combined with other materials to form composites with UV shielding properties [21]. Recently, Yang et al. prepared highly transparent UV shielding protective films by adsorption of iron ions on cellulose nanofibril with carboxyl groups [22]. Unfortunately, the UV-protective film may have low water resistance, as Fe^3+^ washes off easily in water, resulting in reduced UV shielding properties. Meanwhile, Fe^3+^ also causes a dramatic decrease in the mechanical strength of the film. To address these issues, we consider that embedding iron-based MOF into cellulosic films may avoid this defect, as the MOF exists in the form of particles rather than ions. Today, Fe-based MOFs such as MIL-100(Fe), MIL-101(Fe), MIL-53(Fe), MIL-88A(Fe) and MIL-88B(Fe) have been used in various applications due to their non-toxicity, low cost and biocompatibility, easy availability of organic linkers and excellent physicochemical properties [23,24,25,26,27,28,29]. Among them, MIL-88A(Fe), consisting of fumarate and Fe(III), can be synthesized at room temperature in green solvents and has high chemical stability in water. Although various applications of MIL-88A(Fe) have been reported so far, it has not yet been used for efficient UV shielding.

In this work, a transparent carboxymethylated cellulose hydrogel platform was prepared from filter paper cellulose fibers. Then, based on this platform, flexible, biodegradable, and UV-blocking nano-MIL-88A(Fe)@carboxymethylated cellulose films (M(Fe)CCFs) were fabricated by in situ synthesis of nano-MIL-88A(Fe) and natural drying. The hypothesis is that nano-MIL-88A(Fe) particles are uniformly distributed in the cellulose matrix and can impart excellent and durable UV-blocking functionality to cellulose films while maintaining high transmittance (>80%) and low haze (<5%). The UV blocking properties under certain harsh environmental conditions such as high temperature, UV irradiation, acidic or alkaline conditions are explored, which may open up wider prospects for the application of composite films. Our proposed methodology is green, easy to operate, efficient, simple both in the post-treatment stage and purification and in line with the principles of green chemistry, as well as sustainable development.

## 2. Materials and Methods

### 2.1. Materials and Reagents

Quantitative filter paper was obtained from Hangzhou Special Paper Co., Ltd. (Hangzhou, China). Lithium hydroxide (LiOH) and urea were purchased from Shanghai Chemical Reagent Co., Ltd. (Shanghai, China). Sodium hydroxide (NaOH) was purchased from Xiya Chemical Industry Co., Ltd. (Shandong, China). Fumaric acid, glycerol and Ferric chloride hydrate (FeCl_3_·6H_2_O) were provided by Fuyu Fine Chemical Co., Ltd. (Tianjin, China). Sodium chloroacetate was provided by Macklin Biochemical Co., Ltd. (Shanghai, China).

### 2.2. Fabrication of Carboxymethylated Cellulose Fibers

Filter paper was ground to powder using a grinder. Then, 10 g of powdery filter paper was added to a 250 mL sodium chloroacetate solution (1 M) prepared in 15% (*w*/*v*) sodium hydroxide at 50 °C for 2 h. The product (carboxymethylated cellulose fibers) was obtained by filtration and washed with tap water to remove residues until neutral pH. Carboxymethylated cellulose fibers then were dried at 65 °C for subsequent experiment and characterization. The methylene blue method was used to determine the content of the carboxyl group of original and carboxymethylated cellulose fibers [30].

### 2.3. Fabrication of MIL-88A(Fe)@Carboxymethylated Cellulose Films (M(Fe)CCFs)

The powdery carboxymethylated cellulose fibers were added to the 4.6 wt% of LiOH/15.0 wt% of aqueous urea medium and then frozen in a −20 °C freezer for 12 h. The mixture was thawed at room temperature and stirred vigorously to form 4 wt% cellulose solution. Subsequently, the cellulose solution was sonicated in an ice water bath for 20 min to remove air bubbles. The de-bubbled cellulose solution was cast on a glass mold, anhydrous ethanol was added, and after regeneration, carboxymethylated cellulose hydrogel (thickness approx. 1.2 mm) was obtained and thoroughly washed with water. A given amount of FeCl_3_·6H_2_O (Table S1) was solubilized in 75 mL distilled water. The carboxymethylated cellulose hydrogels were added to the FeCl_3_ solution, and then soaked in 25 °C for 3 h. A given amount of fumaric acid (Table S1) was solubilized in 75 mL of anhydrous ethanol. The carboxymethylated cellulose hydrogel adsorbed with Fe^3+^ was added to the fumaric acid ethanol solution and then reacted at 25 °C for 24 h. The MIL-88A(Fe)@carboxymethylated cellulose hydrogels were washed with anhydrous ethanol and distilled water, and then soaked in 5 wt% glycerol solution for 10 min. Finally, the MIL-88A(Fe)@carboxymethylated cellulose hydrogels were fixed to the glass plate to prevent contraction and dried at 25 °C. The MIL-88A(Fe)@carboxymethylated cellulose films (0.12 mm) were obtained, called as M(Fe)CCF1, M(Fe)CCF2 and M(Fe)CCF3. The content of MIL-88A(Fe) in M(Fe)CCFs was determined by weight method. A known weight of M(Fe)CCF was immersed in 50 mL of an aqueous solution containing hydrochloric acid (pH = 1) and sonicated to completely dissolve MIL-88A(Fe). The samples were then removed, washed several times with distilled water and then dried under a vacuum. The weight of the sample before and after was recorded and the weight difference was attributed to the content of MIL-88A(Fe). Pure carboxymethylated cellulosic film (CCF, 0.12 mm) was also prepared.

For comparison purposes, pure MIL-88A(Fe) powder and carboxymethylated cellulose film-Fe^3+^ (CCF-Fe^3+^) were prepared. Powdery MIL-88A(Fe) was synthesized in the same way as M(Fe)CCFs, except for the addition of carboxymethylated cellulose hydrogel. The synthesis of CCF-Fe^3+^ was as follows: Carboxymethylated cellulose hydrogels were added into FeCl_3_ solution (Table S1) for 3 h, washed with distilled water and dried at 25 °C. The carboxymethylated cellulose-Fe^3+^ composite films with a thickness of about 0.12 mm were obtained as CCF-Fe^3+^1, CCF-Fe^3+^2 and CCF-Fe^3+^3.

### 2.4. Environmental Resistance Test

The effect of certain extreme conditions such as UV exposure, pH or temperature on the stability of UV blocking ability was investigated. M(Fe)CCF1 was placed in the oven at different temperatures (120 °C and 150 °C), or under UV light (365 nm, 300 W), or under a solution with different pH values (4.2, 6.8 and 9.2) for 6 h. The UV-Vis transmittance curves were measured.

### 2.5. Biodegradability Test

The enzymatic degradation method was used to measure the biodegradability of M(Fe)CCFs [31]. The experiment was performed with cellulase (0.7 U/mg, Sigma-Aldrich, St. Louis, MI, USA) in acetate buffer (pH = 5) at 37 °C. Approximately 40 mg of M(Fe)CCFs were soaked in 10 mL of cellulase-acetate buffer solution (0.1 mg/mL) for a certain interval. Then, the M(Fe)CCFs were washed, and vacuum dried at 35 °C. The result of enzymatic degradation was expressed as the percentage of weight loss,

*Weight loss* (%) = (*m*_0_ − *m*_t_)/*m*_0_ × 100 (1)

where *m*_0_ and *m*_t_ are the masses of M(Fe)CCFs before and after enzymatic degradation tests, respectively.

### 2.6. Characterization

Thermo Scientific Nicolet iS5 FTIR spectrometer (Nicolet 6700, Waltham, MA, USA) was used to collect Fourier transform infrared (FTIR) spectra in the range of 4000–500 cm^−1^. X-ray diffraction (XRD) patterns were collected on a D/max 2200PC X-ray diffractometer (Rigaku Corporation, Tokyo, Japan). ESCALAB 250Xi (Thermo Fisher Scientific, Waltham, MA, USA) with Al-Kα radiation source was used to conduct an X-ray photoelectron spectroscopy (XPS) test. Prior to FTIR, XRD and XPS tests, all films are ground to a powder using an A11 basic grinder (IKA, Staufen, Germany). The surface and cross-sectional morphologies of the films were observed using a scanning electron microscope (SEM, Hitachi S4800, Tokyo, Japan), and the elemental composition was analyzed using an energy dispersive X-ray spectrometer (EDS). The STA449 F3 equipment was used to perform thermogravimetric analysis (TGA) at a heating rate of 10 °C-min^−1^ under a nitrogen atmosphere. The UTM2203 universal testing machine (Shenzhen SUNS Technology Stock Co. Ltd., Shenzhen, China) was used to test tensile stress–strain curves according to ISO527-3-1995 (E) at a speed of 2 mm min^−1^. A UV-Vis spectrophotometer equipped with an integrating sphere (TU-1950, Beijing, China) was used to test optical transmittance and haze. The haze was tested and calculated according to GBT 2410-2008.

## 3. Results

### 3.1. Structure and Morphology of M(Fe)CCFs

**Scheme 1:**
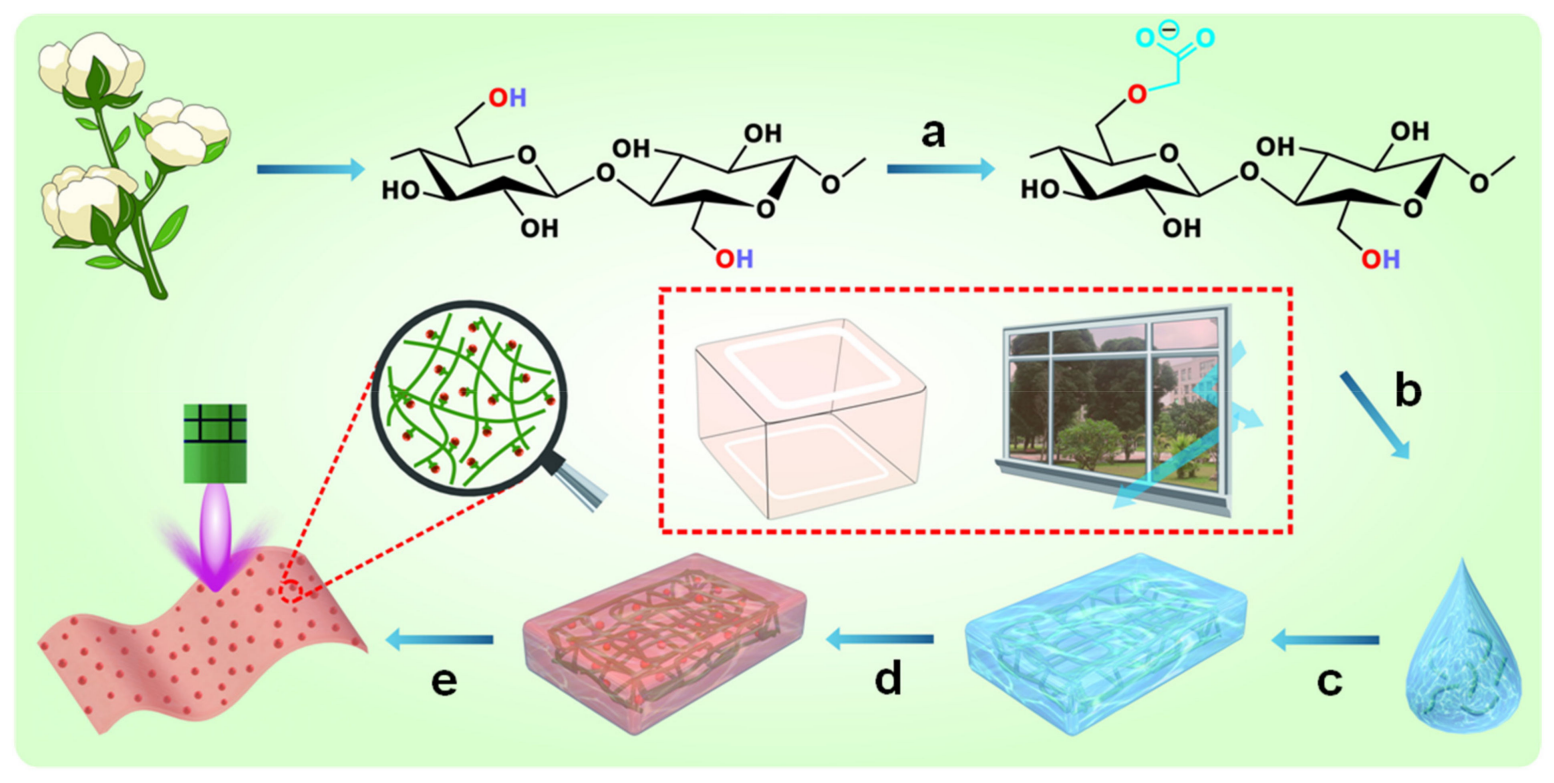
Schematic illustration of concept of fabricating M(Fe)CCFs based on cellulose and nano-MIL-88A(Fe) for UV-blocking. (a) Carboxymethylation of cellulose; (b) Dissolution of cellulose; (c) Formation of carboxymethylated cellulose hydrogel; (d) Formation of composite hydrogel by in situ synthesis of nano-MIL-88A(Fe); (e) Formation of clear M(Fe)CCFs by air-drying.

The concept of converting cellulose and nano-MIL-88A(Fe) into a UV-blocking composite film is schematically proposed in Scheme 1. In order to improve the transparency and reduce the haze of the film material, and to further improve the bonding strength of the nano-MIL-88A(Fe) to the cellulose, carboxymethylated cellulose fibers were prepared by the classical carboxymethylation process. Carboxymethylated cellulose fibers were successfully prepared by the etherification reaction of sodium chloroacetate with cellulose using NaOH as a catalyst. After the carboxymethylation modification, the content of carboxyl groups increased from 5.25 ± 0.12 mmol/kg of original cellulose fibers to 165.34 ± 1.65 mmol/kg of carboxymethylated cellulose fibers. Carboxymethylated cellulose hydrogel was obtained by dissolving and regenerating the carboxymethylated cellulose fibers. MIL-88A (Fe) nanoparticles were synthesized in nanopores of carboxymethylated cellulose hydrogels at room temperature using in situ synthesis strategy in a water/alcohol system. The whole procedure did not involve the use of toxic reagents and solvents and was carried out only in the aqueous or aqueous/alcohol phase.

The interaction between nano-MIL-88A(Fe) and cellulose was analyzed, and the mechanism of the interaction is proposed and shown in Figure S1. After carboxymethylation modification, the cellulose backbone contained not only hydroxyl groups but also a large number of active sites represented by carboxyl groups, which provide binding sites for the anchoring of nano-MIL-88A(Fe) in carboxymethylated cellulose hydrogel. The large number of carboxyl groups on the carboxymethylated cellulose adsorbs Fe^3+^ onto the cellulose surface. Subsequently, the carboxymethylated cellulose hydrogel adsorbed with Fe^3+^ is added to ethanol solution containing fumaric acid, and the Fe^3+^ adsorbed on the cellulose surface forms nano-MIL-88A(Fe) nuclei with fumaric acid. As the reaction time increases, MIL-88A(Fe) nanoparticles are gradually formed and then anchored on the surface of cellulose and within the nanopores. After natural drying, the MIL-88A(Fe) nanoparticles are encapsulated in the composite film.

**Figure 1:**
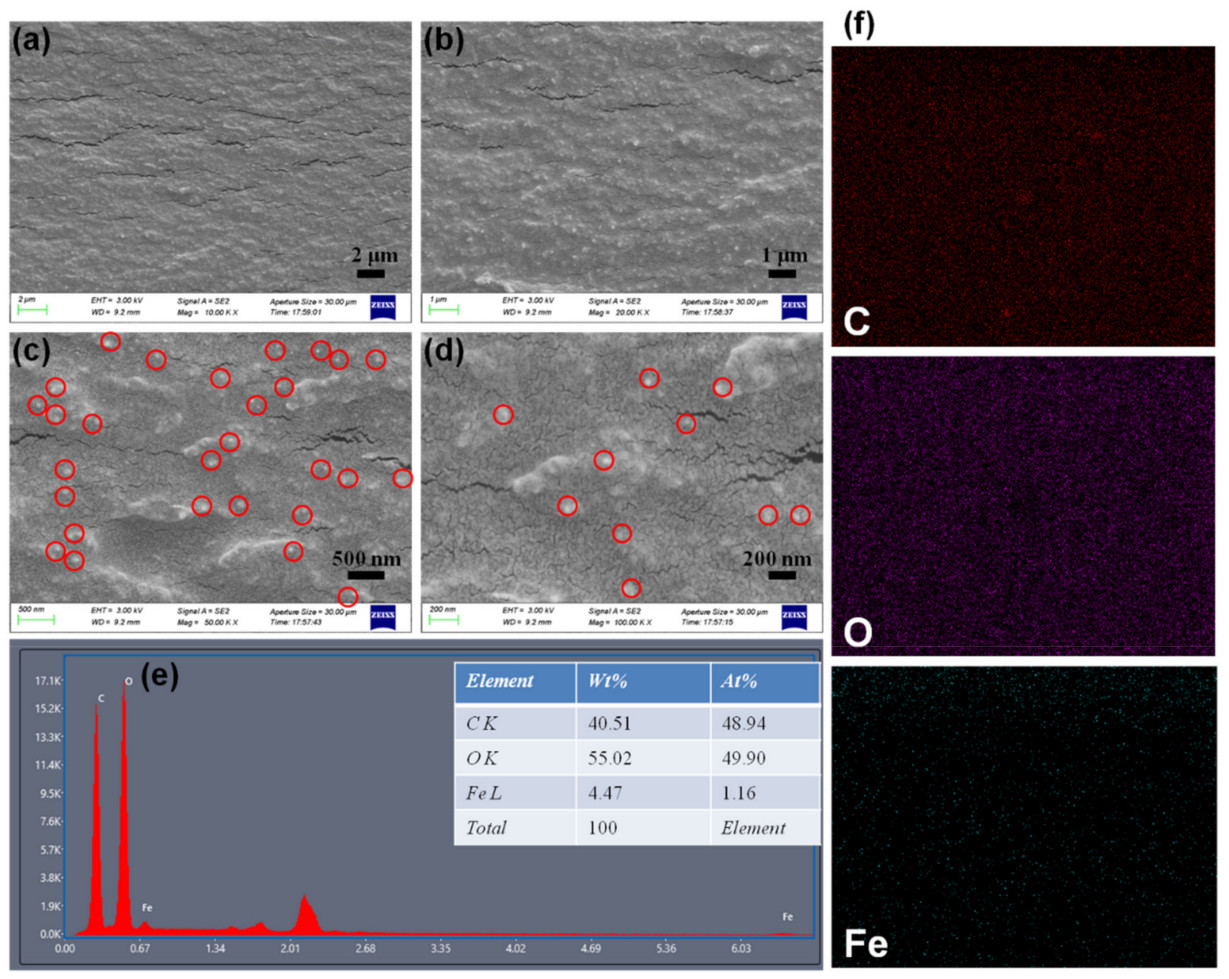
(**a**–**d**) Cross-sectional SEM images of M(Fe)CCF3 under different magnifications; (**e**) EDS spectrum of M(Fe)CCF3; (**f**) Elemental mapping images of M(Fe)CCF3 for carbon, oxygen and iron.

The cross-sectional SEM images of M(Fe)CCFs verify the presence of MIL-88A(Fe) nanoparticles in M(Fe)CCFs. As shown in Figure 1a, there are no obvious MIL-88A(Fe) nanoparticles. In Figure 1b–d, we can see the presence of MIL-88A(Fe), which are tightly embedded in M(Fe)CCF3. The presence of MIL-88A(Fe) can also be observed in the cross-sectional SEM images of M(Fe)CCF1 and M(Fe)CCF2 (Figure S2). Compared with M(Fe)CCF3, less MIL-88A(Fe) can be observed in M(Fe)CCF1 and M(Fe)CCF2. Additionally, the MIL-88A(Fe) nanoparticles are evenly distributed in the cellulose matrix without aggregation, showing that MIL-88A(Fe) is successfully embedded in the nanopores of carboxymethylated cellulose hydrogel. The particle size distribution of MIL-88A(Fe) was detected in M(Fe)CCF3 in the range of 32.5–83.6 nm. Figure 1e shows the results of the EDS spectrum of M(Fe)CCF3. From the EDS spectrum, it can be seen that M(Fe)CCF3 contains C, O and Fe elements, which confirms the formation of MIL-88A(Fe) nanoparticles within M(Fe)CCF3. To further analyze the distribution of each element, the samples were analyzed for elemental distribution and the results are shown in Figure 1f. In M(Fe)CCF3, the uniform distribution of Fe elements was detected, in addition to C and O elements. Figure S3 shows the surface SEM images of M(Fe)CCF3. It can be observed that M(Fe)CCF3 displays a dense and homogeneous surface. Additionally, it can be observed that the surface of M(Fe)CCF3 is smooth with no visible MIL-88A(Fe) nanoparticles. This is attributed to the fact that MIL-88A(Fe) nanoparticles are mainly formed in the cellulose hydrogel nanopores, rather than on the hydrogel surface. Figure S4 shows the three-dimensional (3D) topographic maps of the CCF and M(Fe)CCF3 using atomic force microscopy (AFM). The AFM image of CCF and M(Fe)CCF3 clearly displays a homogeneous and flat surface. The roughness average (Ra) of the CCF and M(Fe)CCF3 was about 7.8 and 8.05 nm, respectively.

**Figure 2:**
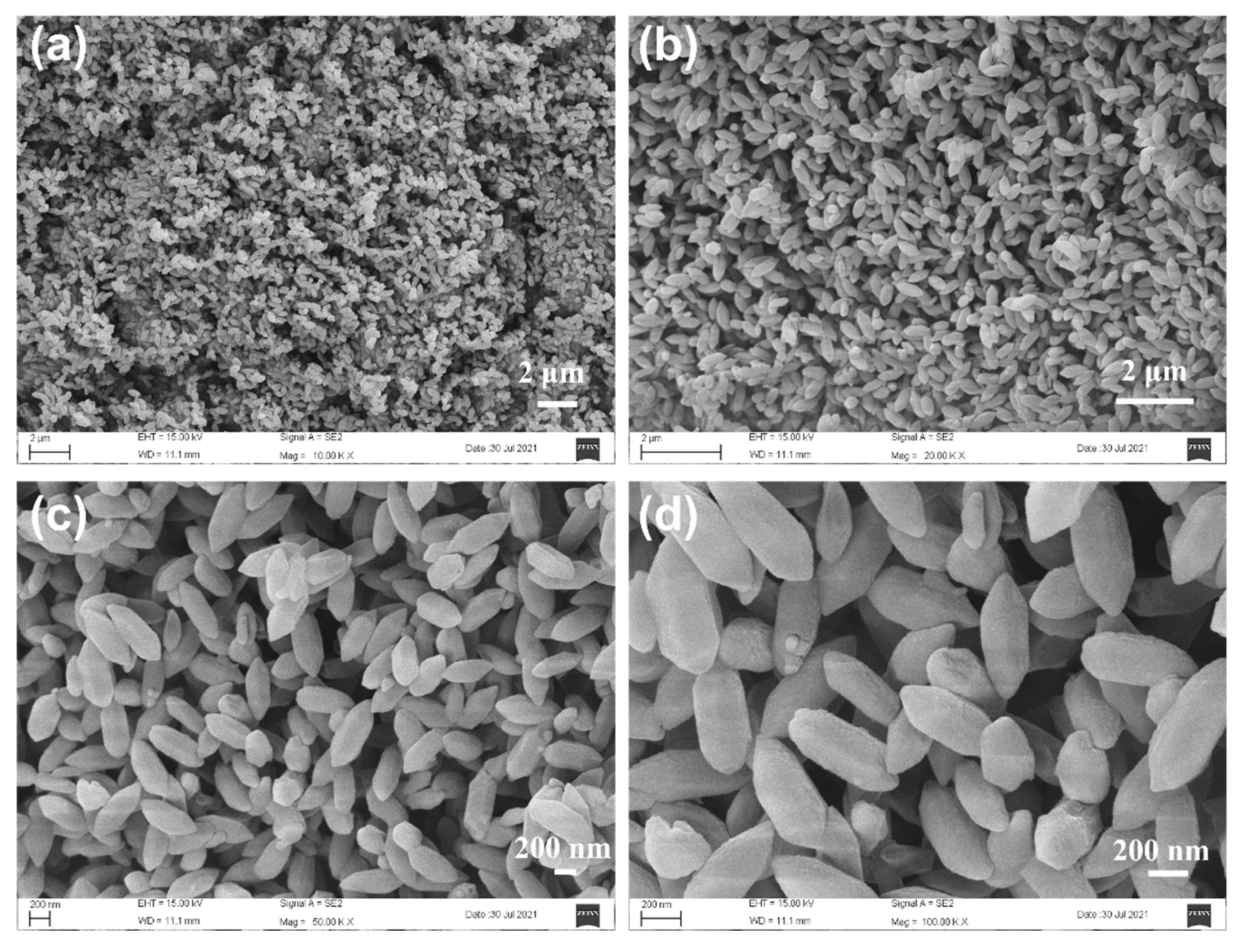
SEM images of MIL-88A(Fe) under different magnifications. (**a**) ×10,000 magnification, (**b**) ×20,000 magnification (**c**) ×50,000 magnification and (**d**) ×100,000 magnification.

The current methods for the synthesis of MIL-88A(Fe) are mostly hydrothermal or solvothermal, both of which require high temperature or organic solvents [32]. Therefore, a simple, low-temperature and green preparation method for MIL-88A(Fe) deserves attention, which not only reduces energy consumption but also facilitates the development and application of MIL-88A(Fe). In this work, a green, simple and room temperature preparation method for MIL-88A(Fe) was used, obtained by reacting an aqueous solution of FeCl_3_ with an ethanolic solution of fumaric acid [33]. As shown in Figure 2, pure MIL-88A(Fe) shows a homogeneous and well-crystallized spindle shape with an average width of 245 nm and an average length of 650 nm. It is noteworthy that the size of MIL-88A(Fe) particles in M(Fe)CCF was much smaller than that of directly synthesized MIL-88A(Fe), and the size of MIL-88A(Fe) also changed its morphology from spindle-shaped to spherical nanoparticles. This is attributed to the “nano-confinement” effect of the nanopores in cellulose hydrogel, which facilitates the preparation of small, uniformly distributed MIL-88A(Fe) nanoparticles.

### 3.2. FTIR Analysis

**Figure 3:**
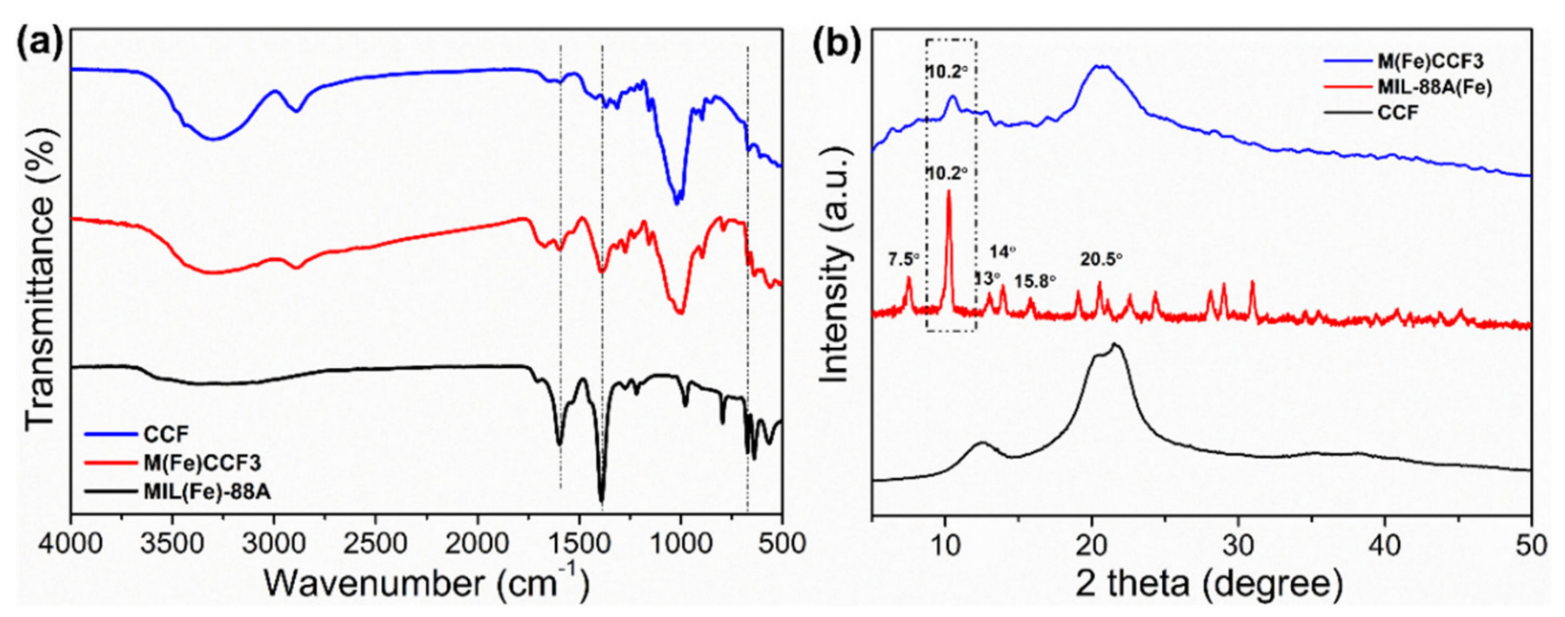
(**a**) FTIR spectra of CCF, MIL-88A(Fe) and M(Fe)CCF3; (**b**) XRD patterns of CCF, MIL-88A(Fe) and M(Fe)CCF3.

The FTIR spectra of CCF, MIL-88A(Fe) and M(Fe)CCF3 are displayed in Figure 3a. For MIL-88A(Fe), the broad absorption band around 3300–3500 cm^−^^1^ is ascribed to the O-H stretching vibration of the water molecule [33]. The characteristic peaks at 1396 cm^−^^1^ and 1603 cm^−^^1^ ascribed to the symmetric and asymmetric vibrational modes of the carboxylate group, respectively, showing the presence of the dicarboxylic acid ligand in the MIL-88A(Fe) [34,35]. The characteristic peak at 570 cm^−^^1^ is ascribed to the Fe−O vibration [33]. For CCF, a new characteristic peak at 1593 cm^−^^1^ is ascribed to the introduction of carboxyl groups on the backbone of cellulose [36]. The characteristic peaks of M(Fe)CCF3 at 569 cm^−^^1^, 1396 cm^−^^1^ and 1603 cm^−^^1^ were derived from MIL-88A(Fe), confirming the successful synthesis of MIL-88A(Fe) within M(Fe)CCF3. No significant characteristic peaks for MIL-88A(Fe) were observed in the FTIR spectra of M(Fe)CCF1 and M(Fe)CCF2 (Figure S5a), which may be due to the low content of MIL-88A (Fe) or the fact that MIL-88A(Fe) is embedded in the cellulose matrix and therefore less exposed after grinding.

### 3.3. XRD Analysis

The XRD patterns of MIL-88A(Fe), CCF and M(Fe)CCF3 are displayed in Figure 3b. In the XRD pattern of MIL-88A(Fe), 2θ = 7.5°, 10.2°, 13.0°, 14°, 15.2° and 20.9° are the characteristic diffraction peaks of MIL-88A(Fe) [33,37,38]. Similar results were reported by Fu et al., indicating that MIL-88A(Fe) can be synthesized using this green method [33]. CCF has three strong diffraction peaks at 2θ = 12.4°, 20.2° and 22.2° corresponding to (1–10), (110) and (020) crystal plane diffraction of cellulose type II. M(Fe)CCF3 exhibited a strong diffraction peak at 2θ = 10.2°, demonstrating the successful synthesis of MIL-88A(Fe) in cellulose matrix. In the XRD patterns of M(Fe)CCF1 and M(Fe)CCF2, no diffraction peaks of MIL-88A(Fe) were observed, which may be due to the low content of MIL-88A (Fe) (Figure S5b).

### 3.4. XPS Analysis

**Figure 4:**
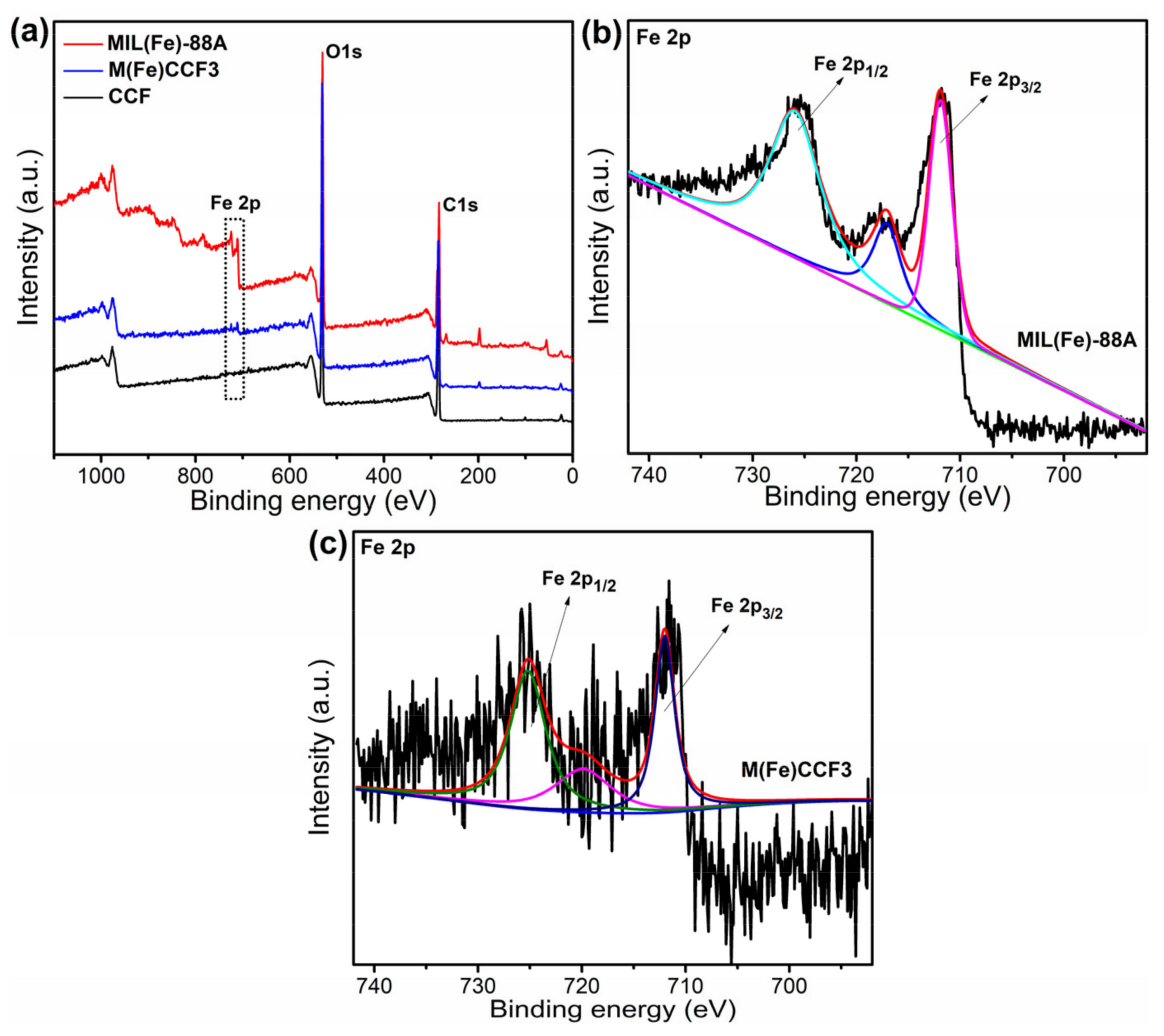
(**a**) The full wide-scan XPS spectra of CCF, MIL(Fe)-88A and M(Fe)CCF3; (**b**,**c**) The high-resolution Fe 2p spectra of the MIL(Fe)-88A and M(Fe)CCF3.

The chemical composition of the samples was analyzed by XPS. XPS spectra of MIL-88A(Fe), CCF, M(Fe)CCF1, M(Fe)CCF2 and M(Fe)CCF3 are shown in Figure 4 and Figure S5c. As shown in Figure 4a, the CCF contains only oxygen (O 1s) and carbon (C 1s) peaks. In the XPS spectra of MIL-88A(Fe), in addition to the peaks of C and O, characteristic peaks of Fe 2p were also observed. No Fe 2p peak was observed in the XPS spectra of M(Fe)CCF1 and M(Fe)CCF2, but it was observed in M(Fe)CCF3. Figure 4b,c show the Fe 2p high resolution spectra of MIL-88A(Fe) and M(Fe)CCF3. The Fe 2p high resolution spectra can be divided into two main peaks, Fe 2p_3/2_ (711.2 eV) and Fe 2p_1/2_ (725.01 eV). The fitted peak at 715.66 eV is usually ascribed to the satellite peaks of Fe 2p_3/2_ and Fe 2p_1/2_ [39]. In summary, these fitted peaks are associated with Fe^3+^, demonstrating the presence of Fe^3+^ in the MIL-88A(Fe) structure. A peak spacing of 13.8 eV exists between the Fe 2p_3/2_ and Fe 2p_1/2_ peaks, which is in perfect agreement with previous references on Fe_2_O_3_, an important feature of Fe(III) present in M(Fe)CCF3 [40,41].

### 3.5. TG Analysis

The thermal stability of CCF and M(Fe)CCFs was investigated by thermogravimetric analysis (TGA), as shown in Figure S6. Both CCF and M(Fe)CCFs exhibited a three-step thermal degradation behavior. It can be observed that the thermal stability of M(Fe)CCFs improves with the increasing content of MIL-88A(Fe). The enhanced thermal stability could be attributed to the presence of MIL-88A(Fe), as it effectively inhibited the volatilization of decomposition products into the gas phase. All M(Fe)CCFs showed higher residue percentages than CCF at 800 °C. This suggests that the introduction of MIL-88A(Fe) improves the thermal stability of M(Fe)CCFs.

### 3.6. UV Light Blocking Performance of M(Fe)CCFs

**Figure 5:**
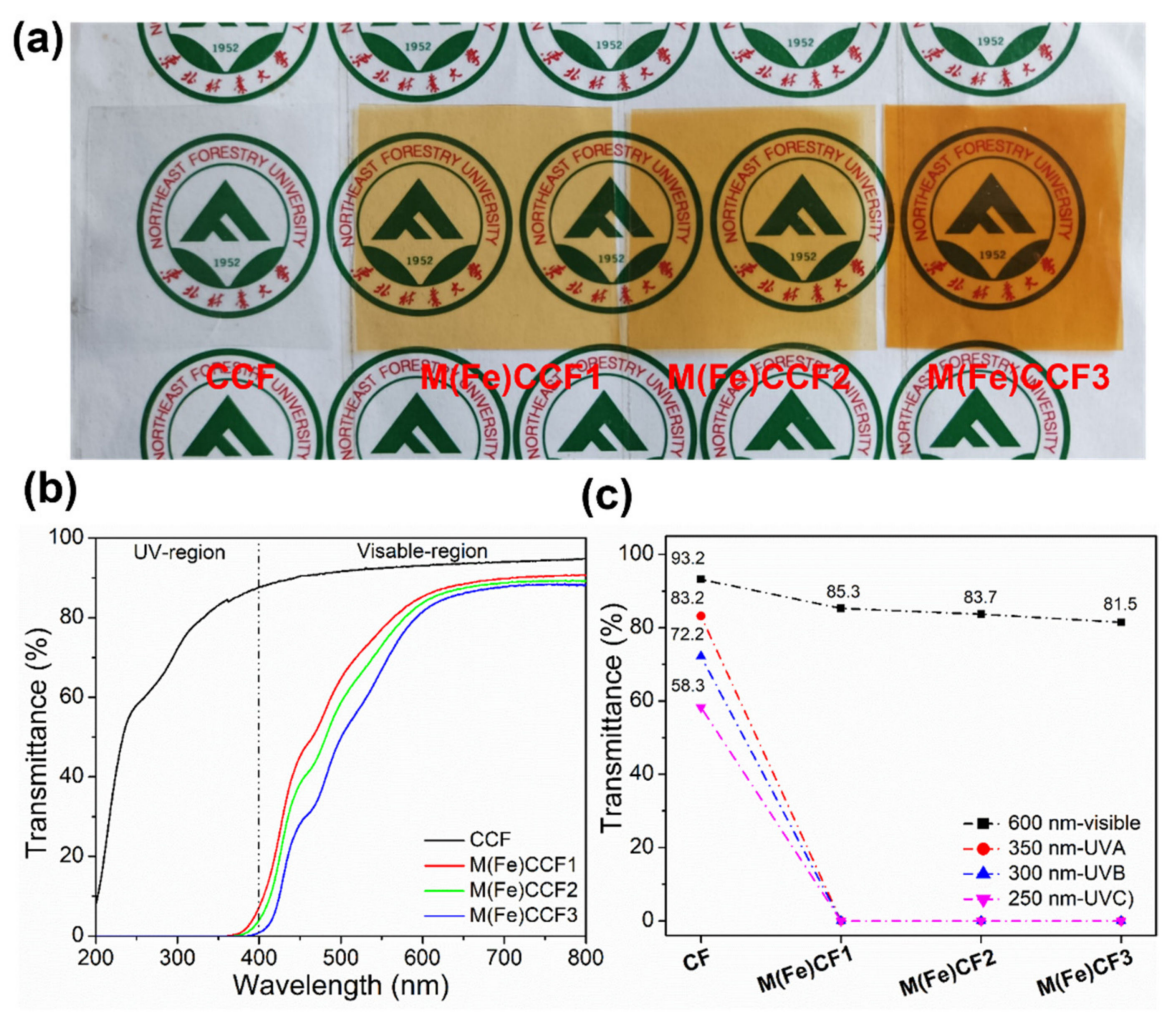
(**a**) Photographs of CCF and M(Fe)CCFs; (**b**) UV-vis light transmittance curves of CCF and M(Fe)CCFs from 200 to 800 nm; (**c**) Transmittance at specific UV light wavelengths.

Figure 5a shows photographs of the CCF and M(Fe)CCFs. The CCF and M(Fe)CCFs are visually highly transparent, indicating that the MIL-88A(Fe) nanoparticles are uniformly distributed in the M(Fe)CCFs. The incorporation of MIL-88A(Fe) nanoparticles into the cellulose matrix resulted in an orange-yellow color of the M(Fe)CCFs, and the color of the M(Fe)CCFs gradually deepened with increasing loading of MIL-88A(Fe) nanoparticles (Figure S7). Figure 5b shows the UV-vis transmittance spectra of CCF and M(Fe)CCFs in the wavelength range of 200–800 nm. The CCF (0.12 mm) has a high transmittance of up to 93.2% at 600 nm. At the same time, it was also observed that CCF still has high transmittance in the UV region of 200–400 nm, which indicates the poor UV shielding ability of CCF. As shown in Figure 5b,c, the UV region was gradually shielded with increasing loading of MIL-88A(Fe) nanoparticles, while the transmittance of M(Fe)CCFs gradually decreased. Despite the low loading of MIL-88A(Fe) nanoparticles, M(Fe)CCF1 still shields all UV rays up to 380 nm (below 380 nm, transmittance was 0). Specifically, M(Fe)CCF1 shields 100% of UVC (200–280 nm), 100% of UVB (280–320 nm) and over 95% of UVA (320–400 nm), while M(Fe)CCF1 exhibits a high transmittance of 85.3% at 600 nm. As MIL-88A(Fe) loading increases, M(Fe)CCF3 shows 100% UVC, 100% UVB and over 99% UVA shielding. Meanwhile, the transmittance of M(Fe)CCF3 at 600 nm remained as high as 81.5% (Figure 5c).

**Figure 6:**
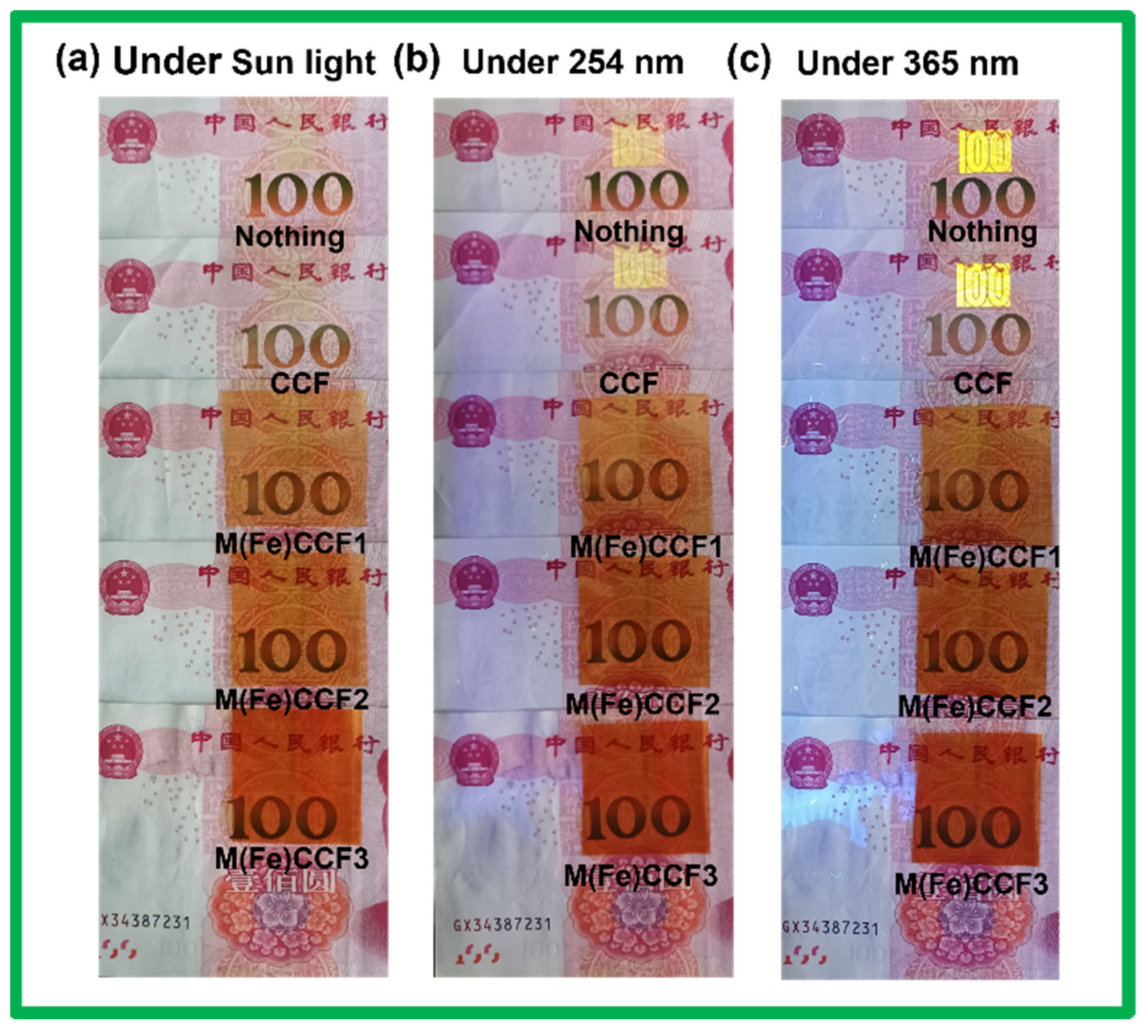
(**a**) Paper money were shielded by nothing, CCF and M(Fe)CCFs and then exposed to sun light. (**b**) Paper money were shielded by nothing, CCF and M(Fe)CCFs and then exposed to UV light (LED, wavenumber of 254 nm); (**c**) Paper money was shielded by nothing, CCF and M(Fe)CCFs and then exposed to UV light (LED, wavenumber of 365 nm).

The excellent UV-shielding capacity of M(Fe)CCFs was also demonstrated by fluorescence pattern of Paper money. As shown in Figure 6a, when Paper money is exposed to sunlight, no fluorescent pattern was observed. Then, we exhibit the fluorescence pattern of Paper money, which fluoresces weakly yellow when exposed to UV light at 254 nm and strongly yellow when exposed to UV light at 365 nm (Figure 6b,c). When CCF was used to cover the fluorescence pattern of Paper money, the yellow fluorescence of the pattern was visible regardless of whether the Paper money was exposed to UV light at 254 nm or 365 nm, indicating that CCF has no UV blocking capability. When the fluorescence pattern of Paper money was covered with M(Fe)CCFs, the yellow fluorescence of pattern was significantly shielded, indicating that M(Fe)CCFs possess superior UV blocking capability.

**Figure 7:**
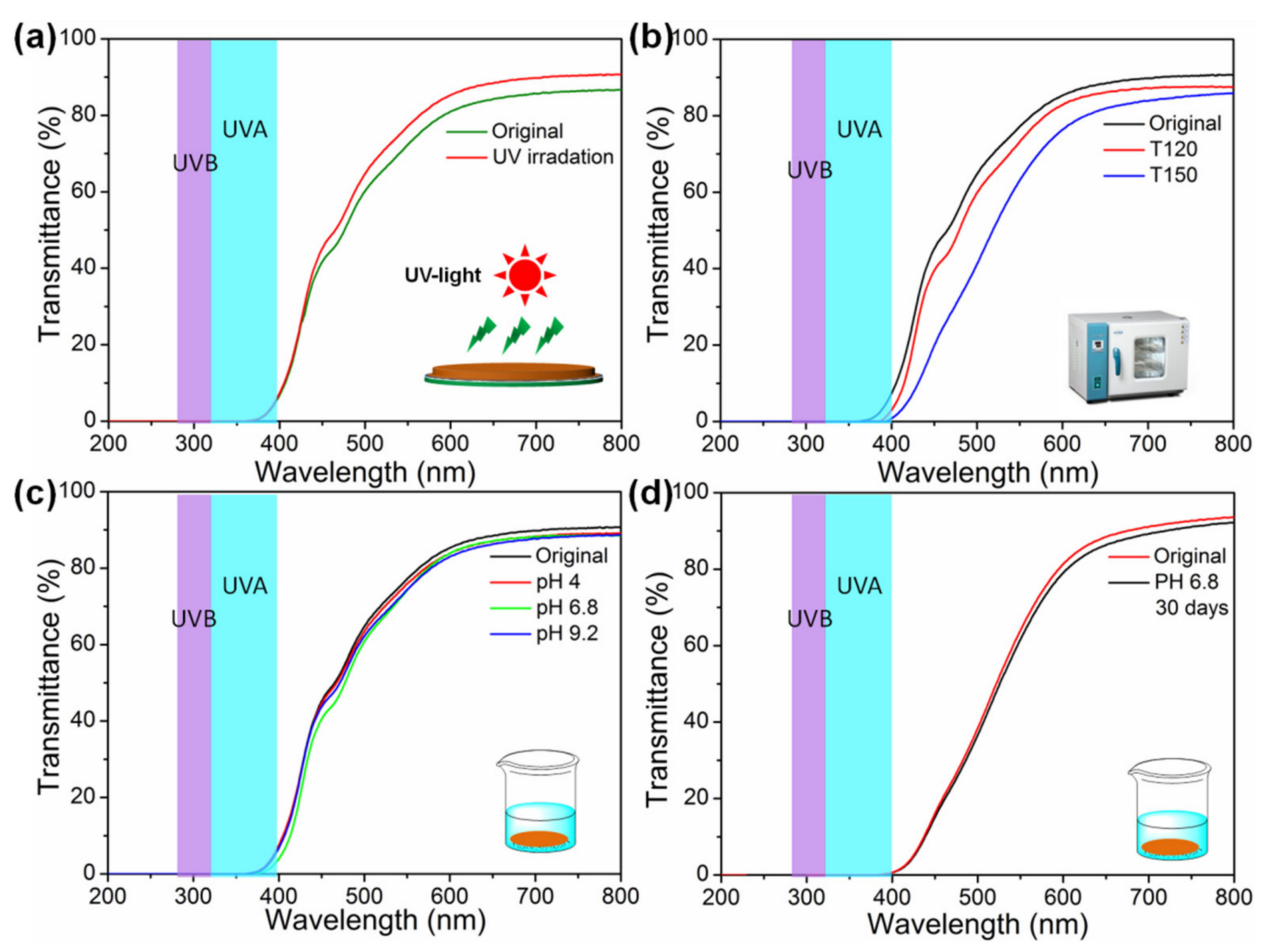
(**a**) Effect of UV irradiation for 6 h on the UV blocking performance of M(Fe)CCF1; (**b**) Effect of heat treatment (120 and 150 °C) for 2 h on the UV blocking performance of M(Fe)CCF1; (**c**) Effect of pH value on the UV blocking performance of M(Fe)CCF1; (**d**) Effect of soaking in water (pH = 6.8) for 30 days on the UV blocking performance of M(Fe)CCF1.

**Figure 8:**
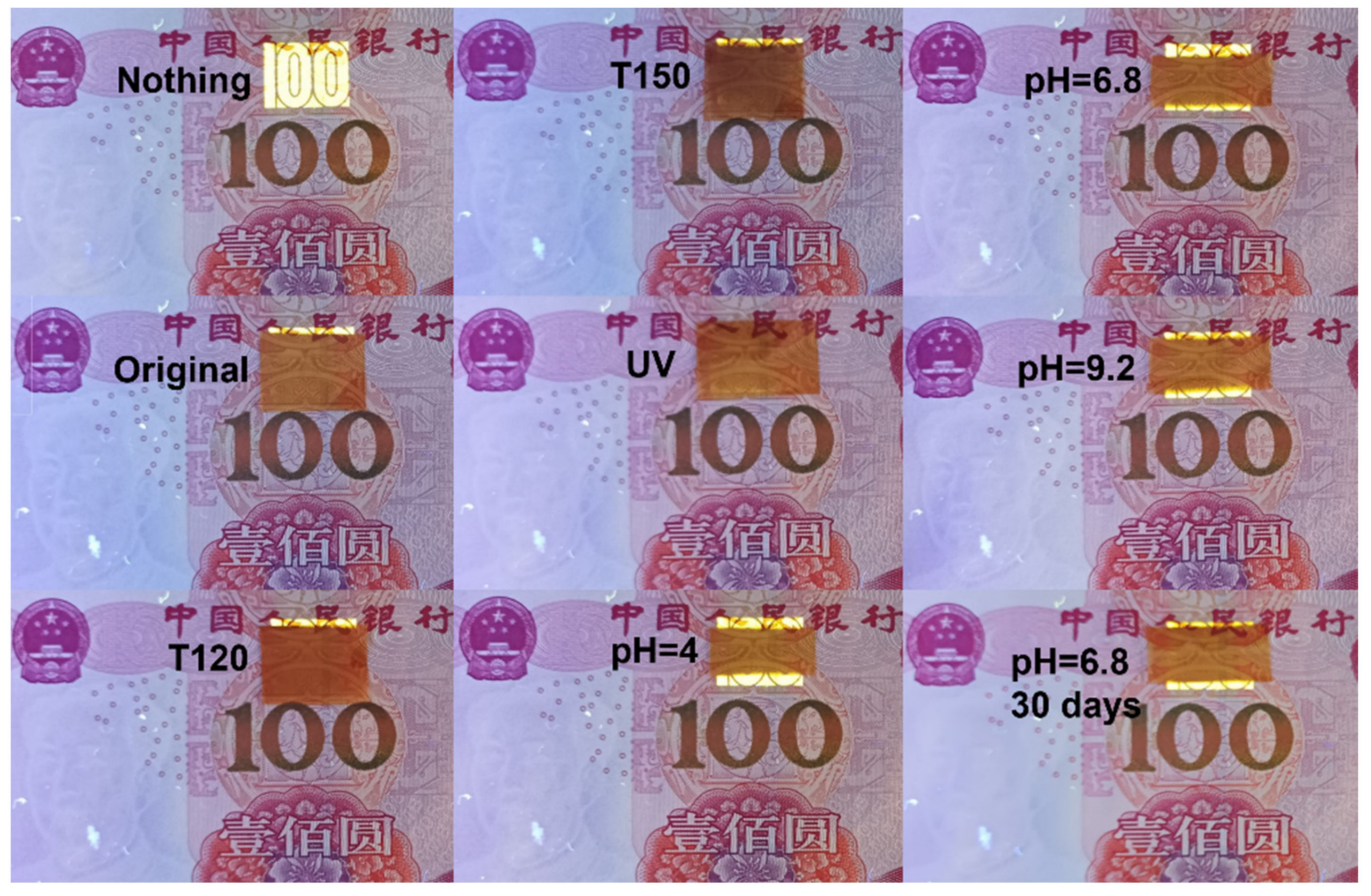
Paper money was shielded by M(Fe)CCF1 treated under different conditions, then exposed to UV light.

Due to the variability of the natural environment, the durability of UV shielding films under extreme conditions is of paramount importance. The effect of UV radiation on the UV shielding stability of the films was investigated as shown in Figure 7a. M(Fe)CCF1 was exposed to 365 nm UV light for 6 h, and the results exhibited that although the transmittance of M(Fe)CCF1 in the visible region decreased slightly, the shielding against UVB and UVA remained stable. The effect of heat treatment at different temperatures on the UV shielding stability is investigated in Figure 7b. Increasing the temperature from 120 °C to 150 °C improves the UV shielding performance of M(Fe)CCF1, especially at 150 °C, with almost complete coverage of UVA and UVB. This phenomenon is mainly due to the deepening of the color, resulting in more intense UV absorption [42]. Yang et al. has reported that the color is the variable that has an effect on UV shielding performance [43]. At the same time, the transmittance of M(Fe)CCF1 decreased with increasing temperature. These results suggest that the heat treatment did not adversely affect the UV shielding stability of M(Fe)CCF1, but rather contributed to the improvement of the UV shielding ability. In addition, the stability of the aqueous environment is also an important factor in assessing the UV shielding stability. M(Fe)CCF1 was immersed in solutions of different pH values (4, 6.8, 9.2) for 6 h, then dried naturally and the UV-Vis transmittance curves were measured (Figure 7c). The results showed that the UVA and UVB shielding properties of M(Fe)CCF1 remained stable in acidic, neutral or alkaline solutions. Additionally, when M(Fe)CCF1 was immersed in an aqueous solution at pH = 6.8 for 30 days and dried naturally, the transmittance curves showed that the UVA and UVB shielding performance of M(Fe)CCF1 was very stable in neutral solutions. This can be attributed to the high water stability of MIL-88A(Fe) and the fact that MIL-88A(Fe) is firmly anchored in the cellulose matrix without structural collapse and detachment (Figure 7d). To perform a visual assessment of the UV shielding stability of M(Fe)CCF1, all treated M(Fe)CCF1 films were placed on the fluorescent pattern of Paper money and then exposed to UV light (365 nm). The treated M(Fe)CCF1 still effectively shielded the anti-counterfeit pattern of Paper money, demonstrating the high UV shielding stability of M(Fe)CCF1 (Figure 8).

**Figure 9:**
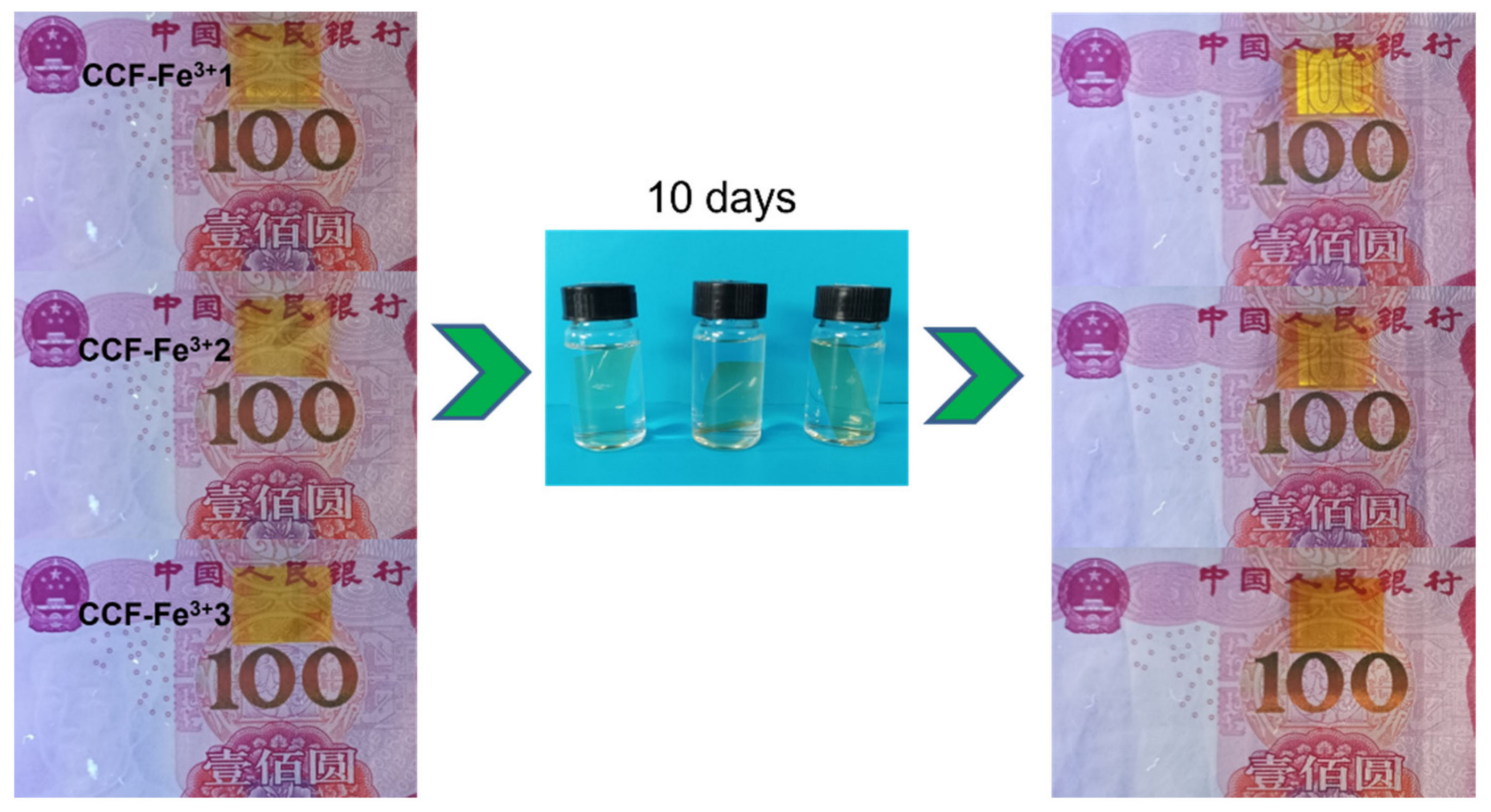
Effect of soaking in water (pH = 6.8) for 10 days on the UV blocking performance of CCF-Fe^3+^ films.

A number of UV absorbers have been incorporated into cellulose-based films to obtain UV shielding capability. Yang et al. reported the preparation of highly transparent TOCN-COOFe^3+^ films with UV shielding capability by adsorbing Fe^3+^ [22]. The results show that after adsorption of Fe^3+^, TOCN-COOFe^3+^ exhibits excellent UV shielding ability. Unfortunately, the UV-protective film may have low water resistance, as Fe^3+^ washes off easily in water, resulting in reduced UV shielding properties. In this work, we compared the UV shielding stability of M(Fe)CCF1 with a carboxymethylated film adsorbed with Fe^3+^ (CCF-Fe^3+^) in an aqueous environment. As shown in Figure 9, the UV shielding ability of the carboxymethylated cellulose films (CCF-Fe^3+^1 and CCF-Fe^3+^2) adsorbed with low Fe^3+^ concentration was significantly weakened after 10 days of immersion in water (pH = 6.8), indicating that Fe^3+^ was gradually released into the solution during the immersion process. In contrast, CCF-Fe^3+^3 still had a high UV shielding capacity, which was due to the adsorption of carboxyl groups partially adsorbed Fe^3+^ on the cellulose surface without being released. Figure 7d and Figure 8 have demonstrated that M(Fe)CCF1 still has excellent UV shielding stability after 30 days of immersion in water, which indicates that MIL-88A(Fe) in the form of nanoparticles is more firmly anchored in the cellulose matrix and has significantly better UV shielding stability than the CCF-Fe^3+^.

**Figure 10:**
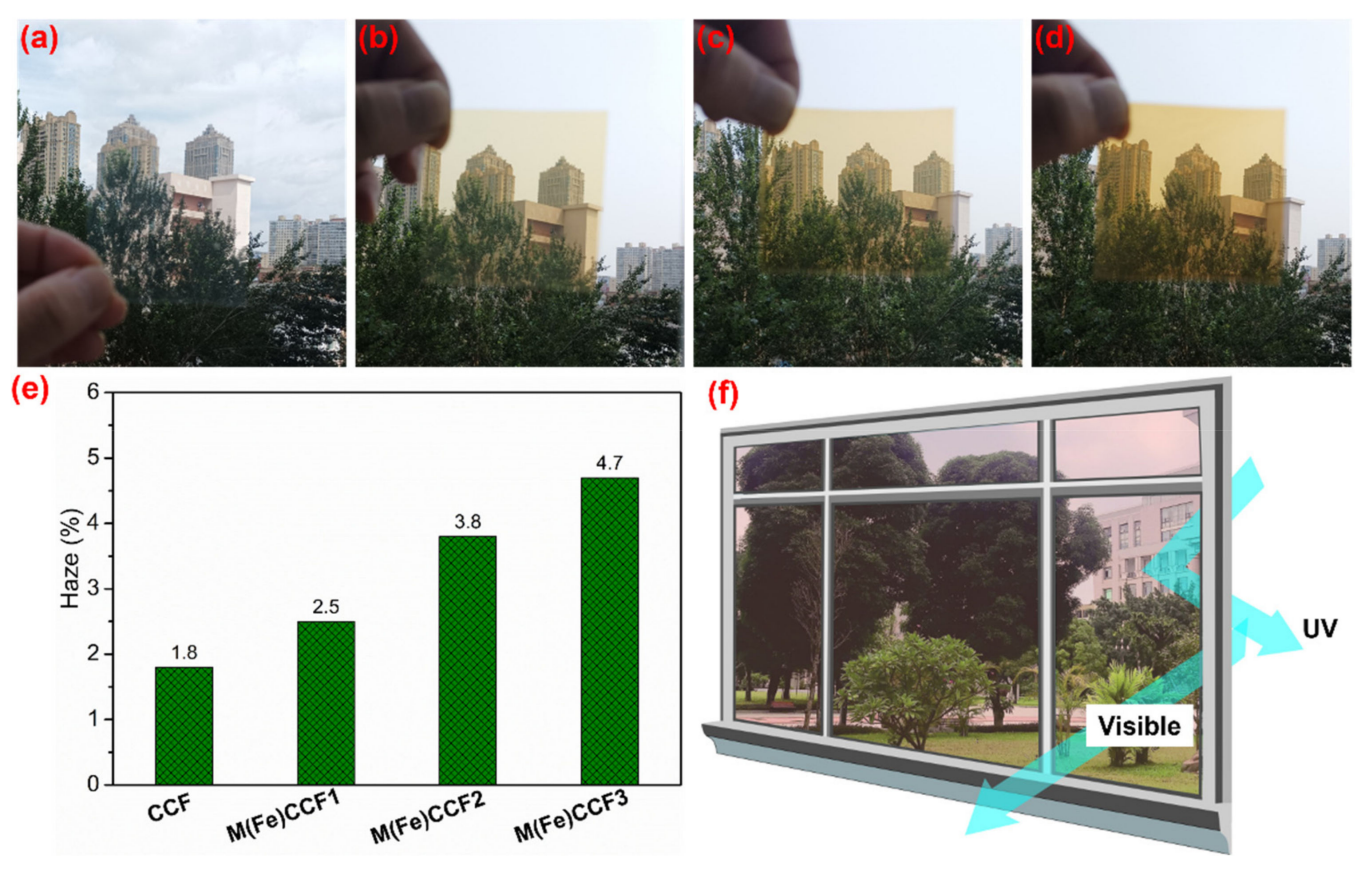
(**a**–**d**) Digital images of CCF, M(Fe)CCF1, M(Fe)CCF2 and M(Fe)CCF3; (**e**) Optical haze of CCF and M(Fe)CCFs at 600 nm; (**f**) Schematic of the potential application of M(Fe)CCF as UV-blocking material in interrupting damage caused by sunlight.

Almost all UV absorbers have a significant impact on the light transmittance and haze of the matrix materials, mainly due to the non-uniform distribution of the UV absorbers in the substrate or their large size and the agglomeration of some small-sized nanoparticles. Interestingly, M(Fe)CCFs not only have superior UV blocking performance but also high transmittance and low haze, which is attributed to the fact that the UV absorber nano-MIL-88A(Fe) is mainly produced in the cellulose hydrogel nanopores and is therefore uniformly distributed. Based on the template effect of the cellulose hydrogel, MIL-88A(Fe) was prepared in a small size. The small size of MIL-88A(Fe) facilitates absorption of UV light and reduces the effect on transmittance and haze. Objects and landscapes viewed through M(Fe)CCFs were also clear, showing that M(Fe)CCFs have low optical haze, greatly extending their range of applications (Figure 10a–d). As shown in Figure 10e,f, the CCF (0.12 mm) not only shows an extremely high transmittance (93.2% at 600 nm), but also a haze of only 1.8% at 600 nm. M(Fe)CCF1 has a high transmittance and can completely shield the entire UVC and UVB region, while shielding over 95% of UVA. M(Fe)CCF1 has excellent UV shielding performance, completely shielding the entire UVC and UVB region, while shielding over 95% of UVA. More importantly, the transmittance of M(Fe)CCF1 is as high as 85.3%, and the haze is only 2.5% (Figure 10f). As the nano-MIL-88A(Fe) content increases, the transmittance of M(Fe)CCF2 decreases slightly and the haze increases. As the nano-MIL-88A(Fe) content continues to increase, M(Fe)CCF3 still has a high transmittance of 81.5% and a low haze of 4.9%. Figure 10f schematically shows the potential application of M(Fe)CCFs as a decorative material to interrupt harmful UV light from sunlight.

### 3.7. Mechanical Property of M(Fe)CCFs

**Figure 11:**
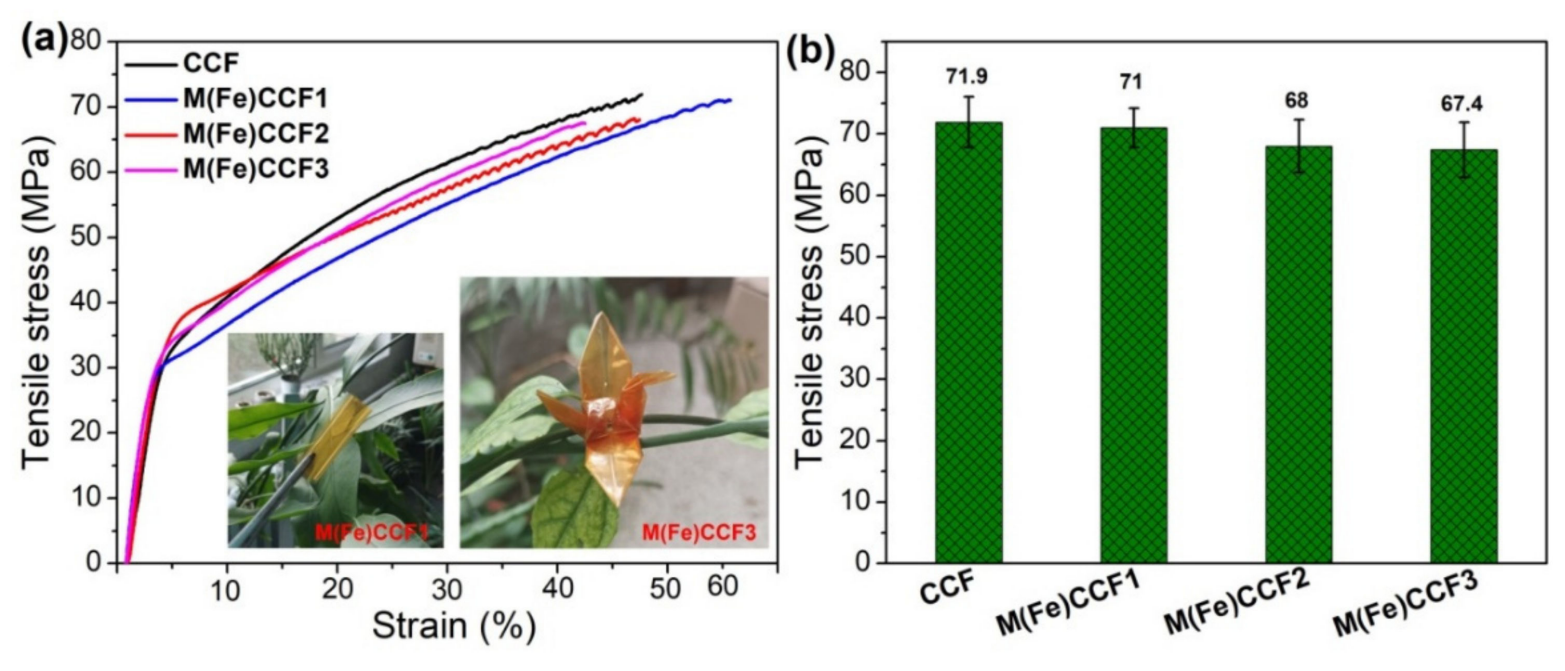
(**a**) The stress–strain curve of CCF and M(Fe)CCFs, the inset is a photo of M(Fe)CCF1 and M(Fe)CCF3, showing good mechanical strength and flexibility; (**b**) Tensile strength of CCF and M(Fe)CCFs.

The stress–strain curves of CCF and M(Fe)CCFs are shown in Figure 11a. The CCF showed a tensile strength of 71.9 MPa, with a decreasing trend for M(Fe)CCFs as the MIL-88A(Fe) content increased (Figure 11b). Compared to the tensile strength of pure CCF, the tensile strength of M(Fe)CCF1 was 71 MPa, a decrease of only 1.25%. The tensile strength of M(Fe)CCF3 was 67.4 MPa, a decrease of 6.26%. Yang et al. prepared transparent UV shielding protective films by introducing Fe^3+^ into nanocellulose [22]. It was found that the introduction of Fe^3+^ significantly reduced the mechanical strength of the films (by 28.50%), which may be due to the disruption of hydrogen bonding by Fe^3+^, resulting in a decrease in the mechanical strength of the films. However, the mechanical strength of M(Fe)CCFs was slightly reduced. The decrease in mechanical strength of M(Fe)CCFs is attributed to the combination of Fe^3+^ with the organic ligand fumaric acid to generate MIL-88A(Fe) nanoparticles, thus reducing the disruption of hydrogen bonds. The M(Fe)CCFs are also easy to handle and resistant to bending and folding, indicating that M(Fe)CCFs have excellent flexibility.

### 3.8. Biodegradability of M(Fe)CCFs

**Figure 12:**
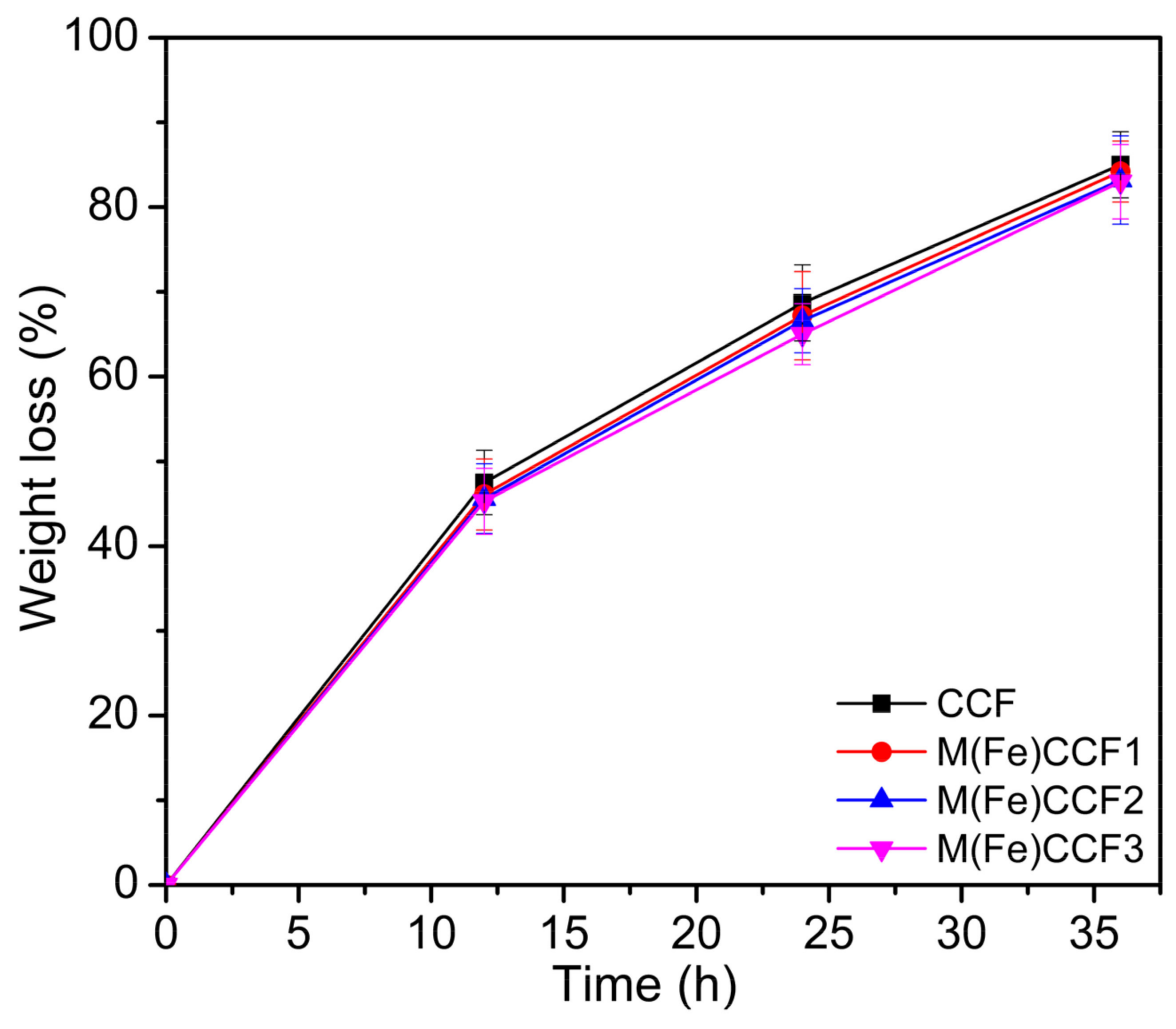
Biodegradation of the CCF and M(Fe)CCFs by the cellulase enzyme.

Polysaccharide rings linked by glycosidic bonds are readily biodegradable by microorganisms and hydrolytic enzymes. The enzymatic degradation expressed as a percentage weight loss is shown in Figure 12. It can be observed that the weight loss values increase with time. After 12 h of incubation with cellulase, the mass losses of CCF, M(Fe)CCF1, M(Fe)CCF2 and M(Fe)CCF3 were 47.5%, 46.1%, 45.6% and 45.3%, respectively. Furthermore, after 36 h of incubation by cellulase, the weight loss of all samples could reach approximately 83%. It can be seen that the presence of MIL-88A(Fe) nanoparticles hardly affected the degradation of the composite films by cellulase. These results demonstrate that both the CCF and M(Fe)CCFs prepared in this study exhibit excellent biodegradability.

## 4. Conclusions

In this work, a flexible and biodegradable cellulose film with comprehensive properties, including high optical transmittance, low haze, UV blocking, and good mechanical strength, was facilely prepared. First, a transparent carboxymethylated cellulose hydrogel platform was prepared from filter paper cellulose fibers. Subsequently, carboxymethylated cellulose gel acts as a nanoreactor to promote growth and anchorage of nano-MIL-88A(Fe). Finally, clear cellulose films were obtained by natural drying. The uniform distribution of nano-MIL-88A(Fe) in M(Fe)CCFs was confirmed by SEM micrographs. The uniform distribution of nano-MIL-88A(Fe) imparts M(Fe)CCF’s excellent UV shielding performance. The pure CCF has high optical transmittance (93.2%) and low haze (1.8%). The introduction of nano-MIL-88A(Fe) endowed M(Fe)CCF’s superior UV-shielding ability, while retaining high transmittance (81.5–85.3%) and low haze (2.5–4.9%). M(Fe)CCFs showed stable UV blocking performance under UV irradiation, high temperature, acidic or alkaline conditions. Meanwhile, the UV-shielding ability of M(Fe)CCFs did not deteriorate even after 30 days of immersion in aqueous solution, providing films with a long-term use capacity. Compared to films made from synthetic polymers, these films are non-toxic, environmentally friendly and biodegradable, reducing the accumulation of microplastics. These films have potential applications in fields such as packaging and flexible electronics.

## Data Availability

Not applicable.

## Supplementary Materials

The following supporting information can be downloaded at: https://www.mdpi.com/article/10.3390/nano12111891/s1, Table S1: Reagents molar amount, reaction time and temperature used for samples; Figure S1: Reaction mechanism describing the formation of MIL-88A(Fe) into carboxymethylated cellulose gel; Figure S2: Cross-sectional SEM images of M(Fe)CCF1 and M(Fe)CCF2. Figure S3: Surface SEM images of M(Fe)CF3 under different magnifications; Figure S4: AFM images of pure CCF and M(Fe)CCF3; Figure S5: (a) FTIR spectra of M(Fe)CCF1 and M(Fe)CCF2, (b) XRD patterns of M(Fe)CCF1 and M(Fe)CCF2; (c) The full wide-scan XPS spectra of M(Fe)CCF1 and M(Fe)CCF2. Figure S6: Thermogravimetric curves of CCF and M(Fe)CCFs. Figure S7: Deposition ratio of MIL-88A(Fe) into M(Fe)CCFs.

## References

[1] Olson E., Li Y., Lin F.-Y., Miller A., Liu F., Tsyrenova A., Palm D., Curtzwiler G.W., Vorst K.L., Cochran E. (2019). Thin biobased transparent UV-blocking coating enabled by nanoparticle self-assembly. ACS Appl. Mater. Interfaces.

[2] Sun L., Li L., An X., Qian X. (2021). Nano-metal organic framework for enhanced mechanical, flame retardant and ultraviolet-blue light shielding properties of transparent cellulose-based bioplastics. Polymers.

[3] Ahmed A., Adak B., Bansala T., Mukhopadhyay S. (2019). Green solvent processed cellulose/graphene oxide nanocomposite films with superior mechanical, thermal, and ultraviolet shielding properties. ACS Appl. Mater. Interfaces.

[4] Zhang X.-F., Song L., Wang Z., Wang Y., Wan L., Yao J. (2020). Highly transparent graphene oxide/cellulose composite film bearing ultraviolet shielding property. Int. J. Biol. Macromol..

[5] Cao X., Huang J., He Y., Hu C., Zhang Q., Yin X., Wu W., Li R.K. (2021). Biodegradable and renewable UV-shielding polylactide composites containing hierarchical structured POSS functionalized lignin. Int. J. Biol. Macromol..

[6] Jia P., Ji X., Zheng B., Wang C., Hao W., Han W., Zhang J., Xia G., Ji X., Zhang J. (2022). Eco-friendly and complete recycling of waste bamboo-based disposable paper cups for value-added transparent cellulose-based films and paper plastic composites. Polymers.

[7] Sirviö J.A., Ismail M.Y., Zhang K., Tejesvi M.V., Ämmälä A. (2020). Transparent lignin-containing wood nanofiber films with UV-blocking, oxygen barrier, and anti-microbial properties. J. Mater. Chem. A.

[8] Guo B., Chen W., Yan L. (2013). Preparation of flexible, highly transparent, cross-linked cellulose thin film with high mechanical strength and low coefficient of thermal expansion. ACS Sustain. Chem. Eng..

[9] Yang Q., Fukuzumi H., Saito T., Isogai A., Zhang L. (2011). Transparent cellulose films with high gas barrier properties fabricated from aqueous alkali/urea solutions. Biomacromolecules.

[10] Sadeghifar H., Venditti R., Jur J., Gorga R.E., Pawlak J.J. (2017). Cellulose-lignin biodegradable and flexible UV protection film. ACS Sustain. Chem. Eng..

[11] Wang W., Zhang B., Jiang S., Bai H., Zhang S. (2019). Use of CeO_2_ nanoparticles to enhance UV-shielding of transparent regenerated cellulose films. Polymers.

[12] Ling Z., Wang K., Liu W., Tang W., Yong Q. (2020). Tuning the cellulose nanocrystal alignments for supramolecular assembly of chiral nematic films with highly efficient UVB shielding capability. J. Mater. Chem. C.

[13] Vuoriluoto M., Hokkanen A., Mäkelä T., Harlin A., Orelma H. (2022). Optical properties of an organic-inorganic hybrid film made of regenerated cellulose doped with light-scattering TiO_2_ particles. Opt. Mater..

[14] Rabani I., Lee S.-H., Kim H.-S., Yoo J., Hussain S., Maqbool T., Seo Y.-S. (2021). Engineering-safer-by design ZnO nanoparticles incorporated cellulose nanofiber hybrid for high UV protection and low photocatalytic activity with mechanism. J. Environ. Chem. Eng..

[15] Emam H.E., Abdelhameed R.M. (2017). Anti-UV radiation textiles designed by embracing with nano-MIL(Ti, In)–metal organic framework. ACS Appl. Mater. Interfaces.

[16] Li G.P., Cao F., Zhang K., Hou L., Gao R.C., Zhang W.Y., Wang Y.Y. (2020). Design of anti-UV radiation textiles with self-assembled metal–organic framework coating. Adv. Mater. Interfaces.

[17] Yang Y., Huang W., Guo Z., Zhang S., Wu F., Huang J., Yang H., Zhou Y., Xu W., Gu S. (2019). Robust fluorine-free colorful superhydrophobic PDMS/NH_2_-MIL-125(Ti)@cotton fabrics for improved ultraviolet resistance and efficient oil–water separation. Cellulose.

[18] Yang Y., Zhang S., Huang W., Guo Z., Huang J., Yang H., Ye D., Xu W., Gu S. (2021). Multi-functional cotton textiles design using in situ generating zeolitic imidazolate framework-67 (ZIF-67) for effective UV resistance, antibacterial activity, and self-cleaning. Cellulose.

[19] Lu L., Hu C., Zhu Y., Zhang H., Li R., Xing Y. (2018). Multi-functional finishing of cotton fabrics by water-based layer-by-layer assembly of metal–organic framework. Cellulose.

[20] Zhang K., Yang Z., Mao X., Chen X.-L., Li H.-H., Wang Y.-Y. (2020). Multifunctional textiles/metal−organic frameworks composites for efficient ultraviolet radiation blocking and noise reduction. ACS Appl. Mater. Interfaces.

[21] Bai Y., Zhao Y., Li Y., Xu J., Fu X., Gao X., Mao X., Li Z. (2020). UV-shielding alginate films crosslinked with Fe^3+^ containing EDTA. Carbohydr. Polym..

[22] Yang W., Wang X., Gogoi P., Bian H., Dai H. (2019). Highly transparent and thermally stable cellulose nanofibril films functionalized with colored metal ions for ultraviolet blocking activities. Carbohydr. Polym..

[23] He X., Fang H., Gosztola D.J., Jiang Z., Jena P., Wang W.-N. (2019). Mechanistic insight into photocatalytic pathways of MIL-100(Fe)/TiO_2_ composites. ACS Appl. Mater. Interfaces.

[24] Yan G., Bo Y., Hui Z., Xu Z. (2018). A g-C_3_N_4_/MIL-101(Fe) heterostructure composite for highly efficient BPA degradation with persulfate under visible light irradiation. J. Mater. Chem. A.

[25] Zhang Z., Li X., Liu B., Zhao Q., Chen G. (2016). Hexagonal microspindle of NH_2_-MIL-101(Fe) metal–organic frameworks with visible-light-induced photocatalytic activity for the degradation of toluene. RSC Adv..

[26] Li M., Wang J., Zheng Z., Zheng Y., Li C., Li Z. (2017). Anchoring NaYF4:Yb,Tm Upconversion Nanocrystals on concave MIL-53(Fe) octahedrons for NIR-light enhanced photocatalysis. Inorg. Chem. Front..

[27] Wang J., Wan J., Ma Y., Wang Y., Pu M., Guan Z. (2016). Metal–organic frameworks MIL-88A with suitable synthesis conditions and optimal dosage for effective catalytic degradation of Orange G through persulfate activation. RSC Adv..

[28] Khasevani S.G., Mohaghegh N., Gholami M. (2017). Kinetic study of navy blue photocatalytic degradation over Ag_3_PO_4_/BiPO_4_@ MIL-88B(Fe)@gC_3_N_4_ core@shell nanocomposite under visible light irradiation. New J. Chem..

[29] Xu B., Yang H., Cai Y., Yang H., Li C. (2016). Preparation and photocatalytic property of spindle-like MIL-88B(Fe) nanoparticles. Inorg. Chem. Commun..

[30] Li Z., Hori N., Takemura A. (2020). Synthesis and characterization of Cu-BTC metal–organic frameworks onto lignocellulosic fibers by layer-by-layer method in aqueous solution. Cellulose.

[31] Xia J., Liu Z., Chen Y., Cao Y., Wang Z. (2020). Effect of lignin on the performance of biodegradable cellulose aerogels made from wheat straw pulp-LiCl/DMSO solution. Cellulose.

[32] Liao X., Wang F., Wang F., Cai Y., Yao Y., Teng B.-T., Hao Q., Shuxiang L. (2019). Synthesis of (100) surface oriented MIL-88A-Fe with rod-like structure and its enhanced fenton-like performance for phenol removal. Appl. Catal. B.

[33] Fu H., Song X.X., Wu L., Zhao C., Wang P., Wang C.C. (2020). Room-temperature preparation of MIL-88A as a heterogeneous photo-Fenton catalyst for degradation of rhodamine B and bisphenol a under visible light. Mater. Res. Bull..

[34] Zhang Y., Zhou J., Chen X., Wang L., Cai W. (2019). Coupling of heterogeneous advanced oxidation processes and photocatalysis in efficient degradation of tetracycline hydrochloride by Fe-based MOFs: Synergistic effect and degradation pathway. Chem. Eng. J..

[35] Lin K.-Y.A., Chang H.-A., Hsu C.-J. (2015). Iron-based metal organic framework, MIL-88A, as a heterogeneous persulfate catalyst for decolorization of Rhodamine B in water. RSC Adv..

[36] Li Z., Hori N., Takemura A. (2020). A comparative study of depositing Cu-BTC metal–organic framework onto cellulosic filter paper via different procedures. Cellulose.

[37] Liu N., Huang W., Zhang X., Tang L., Wang L., Wang Y., Wu M. (2018). Ultrathin graphene oxide encapsulated in uniform MIL-88A(Fe) for enhanced visible light-driven photodegradation of RhB. Appl. Catal. B.

[38] Amaro-Gahete J., Klee R., Esquivel D., Ruiz J.R., Jimenez-Sanchidrian C., Romero-Salguero F.J. (2019). Fast ultrasound-assisted synthesis of highly crystalline MIL-88A particles and their application as ethylene adsorbents. Ultrason. Sonochem..

[39] Pang D., Wang C.-C., Wang P., Liu W., Fu H., Zhao C. (2020). Superior removal of inorganic and organic arsenic pollutants from water with MIL-88A(Fe) decorated on cotton fibers. Chemosphere.

[40] Li X., Pi Y., Wu L., Xia Q., Wu J., Li Z., Xiao J. (2017). Facilitation of the visible light-induced Fenton-like excitation of H_2_O_2_ via heterojunction of g-C_3_N_4_/NH_2_-Iron terephthalate metal-organic framework for MB degradation. Appl. Catal. B.

[41] Yu C., Gou L., Zhou X., Bao N., Gu H. (2011). Chitosan–Fe_3_O_4_ nanocomposite based electrochemical sensors for the determination of bisphenol A. Electrochim. Acta.

[42] Wang Q., Du H., Zhang F., Zhang Y., Wu M., Yu G., Liu C., Li B., Peng H. (2018). Flexible cellulose nanopaper with high wet tensile strength, high toughness and tunable ultraviolet blocking ability fabricated from tobacco stalk via a sustainable method. J. Mater. Chem. A.

[43] Yang W., Gao Y., Zuo C., Deng Y., Dai H. (2019). Thermally-induced cellulose nanofibril films with near-complete ultraviolet-blocking and improved water resistance. Carbohydr. Polym..

