# Supplementary material for: Nano-MIL-88A(Fe) Enabled Clear Cellulose Films with Excellent UV-Shielding Performance and Robust Environment Resistance
Source: Nanomaterials (Basel). 2022 May 31;12(11):1891. doi: 10.3390/nano12111891 (PMC9182417; doi:10.3390/nano12111891)
Supplement: Supplementary file 1 [file nanomaterials-12-01891-s001.zip › nanomaterials-1740073-supplementary.pdf]

nanomaterials

Article

Nano-MIL-88A(Fe) Enabled Clear Cellulose Films with Excellent UV-Shielding Performance and Robust Environment Resistance

Lijian Sun, Xianhui An and Xueren Qian *

Key Laboratory of Bio-based Material Science & Technology, Northeast Forestry University, Ministry of Education, Harbin 150040, China; lantian0308@nefu.edu.cn (L.S.); anxianh509@163.com (X.A.)
* Correspondence: qianxueren@nefu.edu.cn; Tel.: +86-13304642918

Citation: Sun, L.; An, X.; Qian, X. Nano-MIL-88A(Fe) Enabled Clear Cellulose Films with Excellent UV-Shielding Performance and Robust Environment Resistance. *Nanomaterials* **2022**, *12*, 1891. https://doi.org/10.3390/nano12111891

Academic Editor: Hirotaka Koga

Received: 9 May 2022
Accepted: 30 May 2022
Published: 31 May 2022

Publisher's Note: MDPI stays neutral with regard to jurisdictional claims in published maps and institutional affiliations.

Copyright: © 2022 by the authors. Licensee MDPI, Basel, Switzerland. This article is an open access article distributed under the terms and conditions of the Creative Commons Attribution (CC BY) license (https://creativecommons.org/licenses/by/4.0/).

Table S1. Reagents molar amount, reaction time and temperature used for samples

Sample	$FeCl_3 \cdot 6H_2O$ (mmol)	fumaic acid (mmol)	time (h)	temperature (°C)
M(Fe)CCF1	1	1	24	25
M(Fe)CCF2	2	2	24	25
M(Fe)CCF3	4	4	24	25

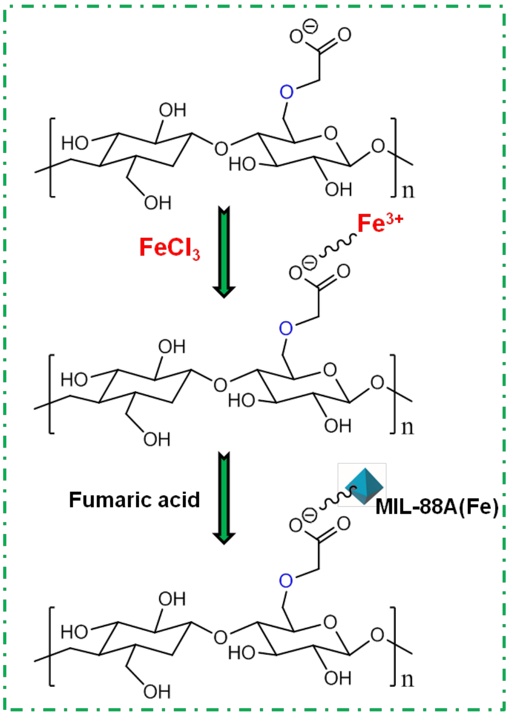

Figure S1. Reaction mechanism describing the formation of MIL-88A(Fe) into carboxymethylated cellulose gel.

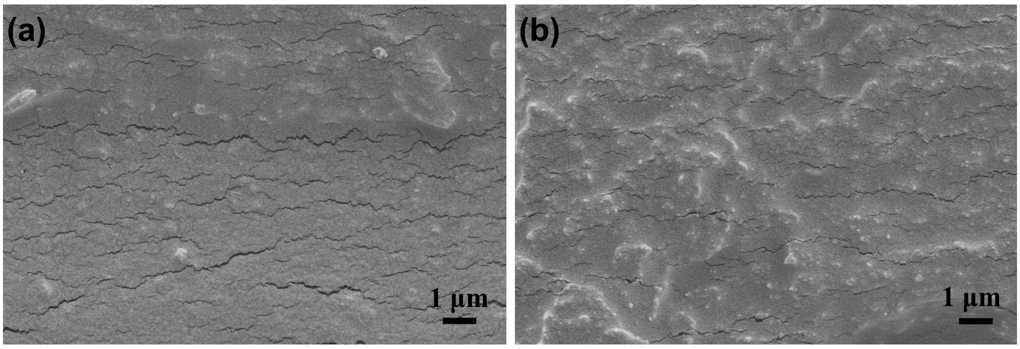

Figure S2. Cross-sectional SEM images of M(Fe)CCF1 and M(Fe)CCF2.

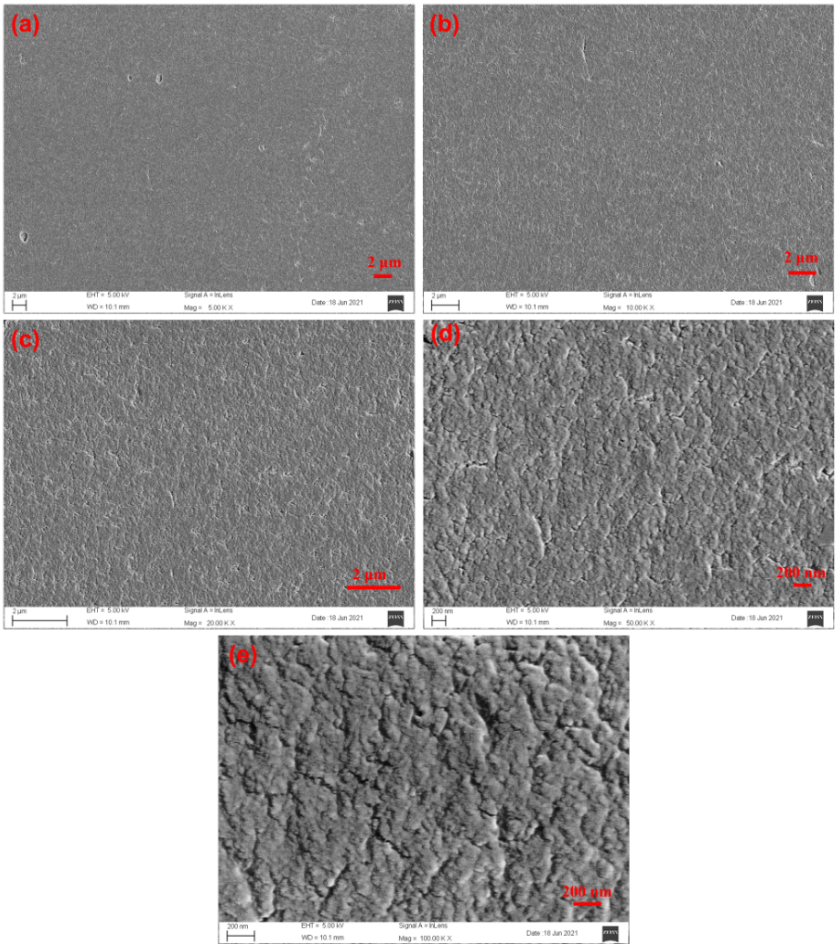

Figure S3. Surface SEM images of M(Fe)CCF3 under different magnifications.

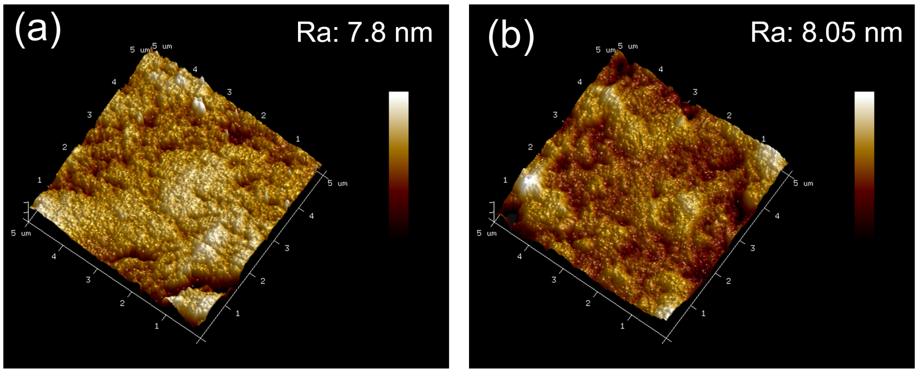

Figure S4. (a) AFM images of pure CCF and (b) M(Fe)CCF3.

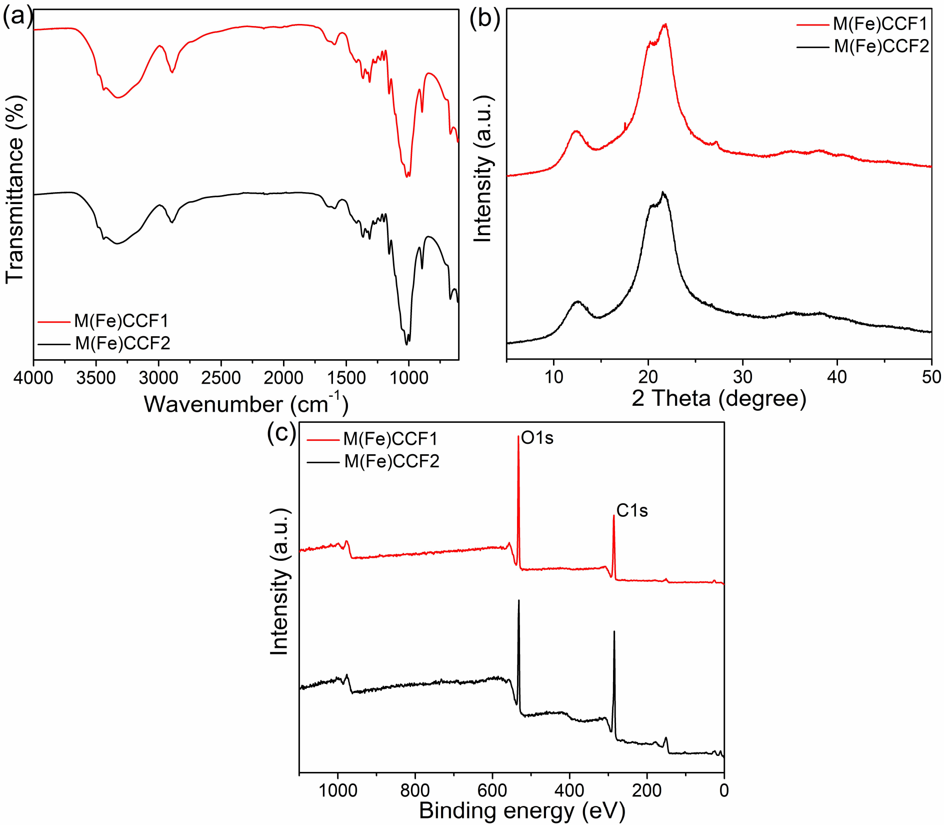

Figure S5. (**a**) FTIR spectra of M(Fe)CCF1 and M(Fe)CCF2; (**b**) XRD patterns of M(Fe)CCF1 and M(Fe)CCF2; (**c**) The full wide-scan XPS spectra of M(Fe)CCF1 and M(Fe)CCF2.

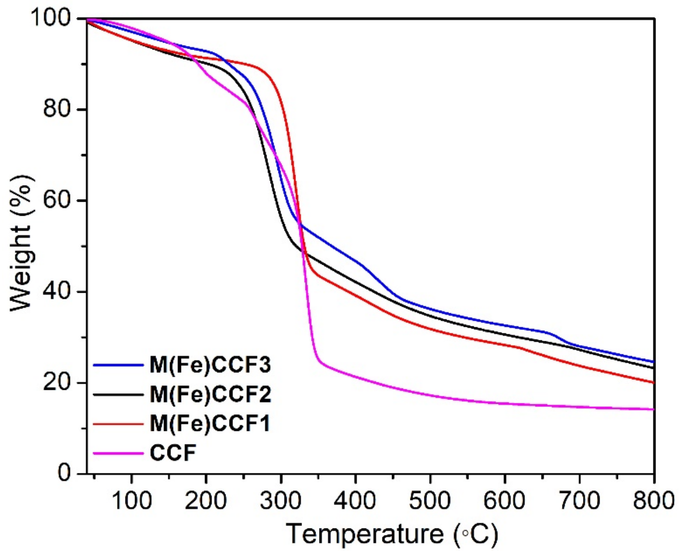

Figure S6. Thermogravimetric curves of CCF and M(Fe)CCFs.

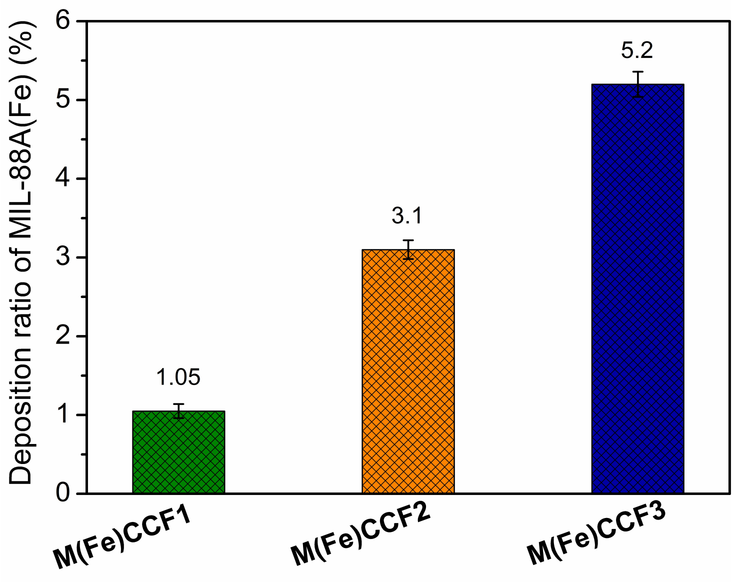

Figure S7. Deposition ratio of nano-MIL-88A(Fe) into M(Fe)CCFs.
